# Multi-Gene Phylogeny and Taxonomy of *Hydnellum* (Bankeraceae, Basidiomycota) from China

**DOI:** 10.3390/jof7100818

**Published:** 2021-09-29

**Authors:** Yan-Hong Mu, Jia-Rui Yu, Ting Cao, Xiang-Hua Wang, Hai-Sheng Yuan

**Affiliations:** 1 CAS Key Laboratory of Forest Ecology and Management, Institute of Applied Ecology, Chinese Academy of Sciences, Shenyang 110164, China; mouyanhong16@mails.ucas.ac.cn (Y.-H.M.); yujiarui18@mails.ucas.ac.cn (J.-R.Y.); caoting215@mails.ucas.ac.cn (T.C.); 2 University of the Chinese Academy of Sciences, Beijing 100049, China; 3 Key Laboratory for Plant Diversity and Biogeography of East Asia, Kunming Institute of Botany, Chinese Academy of Sciences, Kunming 650201, China; xhwang@mail.kib.ac.cn

**Keywords:** nLSU + ITS + SSU + RPB2, stipitate hydnoid fungi, taxonomy, new taxa, Thelephorales

## Abstract

The genus *Hydnellum* is an important group of stipitate hydnaceous fungi which can form ectomycorrhiza with many species of woody plants. In recent decades, the frequency and number of basidiocarps observed in China have been declining significantly. So far, however, we know little about the species diversity of *Hydnellum* in China. In this study, we conducted molecular phylogenetic analyses based on sections of multiple loci, including the large subunit of nuclear ribosomal RNA gene (nLSU), the internal transcribed spacer regions (ITS), the small subunit of nuclear ribosomal RNA gene (SSU) and the second-largest subunit of RNA polymerase II gene (RPB2), as well as morphological studies, of collected samples of *Hydnellum* from China. We also inferred Maximum Likelihood and Bayesian phylogenies for the order Thelephorales from the dataset of the combined nLSU and ITS. This study has revealed the phylogenetic position of *Hydnellum* in the order Thelephorales, and phylogenetically confirmed ten major clades in Thelephorales; Twenty-nine taxa are proposed, described or reported, including 10 new subgenera (*Hydnellum* subgenus *Hydnellum*, subg. *Caesispinosum*, subg. *Croceum*, subg. *Inflatum*, subg. *Rhizomorphum*, subg. *Scabrosum*, subg. *Spongiosum*, subg. *Subindufibulatum*, subg. *Violaceum* and subg. *Zonatum*), 11 new species (*Hydnellum atrorubrum*, *H. atrospinosum*, *H. bomiense*, *H. brunneorubrum*, *H. fibulatum*, *H. granulosum*, *H. inflatum*, *H. rubidofuscum*, *H. squamulosum*, *H. sulcatum* and *H. yunnanense*), 3 newly recorded species (*H. caeruleum*, *H. peckii* and *H. spongiosipes*) and 5 notable specimens (*Hydnellum* sp 1, *H.* sp 2, *H.* sp 3, *H.* sp 4 and *H.* sp 5). A classification system based on the morphological characteristics (especially the hyphal structure types) and molecular analyses is proposed to accommodate most species in *Hydnellum*. The distinguishing characters of the subgenera and the new species with their closely related taxa are discussed. A key to the species of *Hydnellum* from China is provided.

## 1. Introduction

The genus *Hydnellum*, together with *Bankera*, *Phellodon* and *Sarcodon*, are a homogenous group of soil-inhabiting Basidiomycota (with the common characteristic of a hymenophore with a spinulose hymenium) that belongs to the Bankeraceae, Thelephorales [1,2].

All species of Bankeraceae are considered ectomycorrhizal and are associated with woody plants, mainly members of Pinaceae and Fagaceae [3,4,5,6,7], and colonize natural or relatively undisturbed forests [8]. These fungi can absorb organic substances from host plants and also transport nutrients and water from the soil to the plants, which improves the stability of forest ecosystems [9,10]. In addition, some species of *Hydnellum* have important medicinal functions, including cholesterol-lowering, antioxidant, anti-inflammatory, anti-tumor, immune enhancement, etc. [11]. For instance, Lee et al. [12] suggested that *H. concrescens* extracts prevents the expression of NDV-HN glycoprotein on the cell surface by inhibiting the activity of α-glucosidase, thus exhibiting anti-viral function. This can be on par with the health benefit potentials of herbal plant infusions [13].

Due to substantial declines in abundance, they have become the focus of increasing conservation concern [14,15,16]. This is mainly attributed to the effect of habitat loss, aerial pollution, nitrogen deposition and soil acidification [17,18,19,20,21]. Stipitate hydnoid fungi, as symbols for the recent decline of ectomycorrhizal fungi, have been redlisted in, e.g., Norway, Poland, Germany, and the Netherlands [7,14,18,19,22,23,24]. Furthermore, an action plan for 14 rare species of hydnoid fungi has been announced to provide strategic management for their future conservation in the UK [25].

The genus *Hydnellum* is characterized by single to gregarious or coalescent pileate, stipitate basidiocarps, spinous hymenophore, corky to woody, not duplex to duplex, azonate to zonate context, uninflated to inflated generative hyphae, with or without clamp connections, and brown, irregularly ellipsoid to globose, tuberculate basidiospores. Some species display olivaceous or blue-green colours with KOH [1,2,5,26,27]. However, differentiation between closely related species within *Hydnellum* becomes significantly difficult on account of their macromorphological polymorphism caused by growing around obstacles or fusing to other adjacent basidiocarps [2,27]. Therefore, molecular sequence data are very important in identifying them. Molecular evidence has confirmed that *Hydnellum* has a close phylogenetic affiliation with the genus *Sarcodon*, and both genera aggregated in the same clade, named the “*Hydnellum*-*Sarcodon* lineage” [28,29,30]. Furthermore, the phylogenetic analysis of *Hydnellum* and *Sarcodon* according to Baird et al. [27] suggested that the generic limits need reassessment. To revise the generic limits and make genera monophyletic, Larsson et al. [31] moved 12 species from *Sarcodon* to *Hydnellum*, resulting in the generic circumscription of *Hydnellum* being amended. Morphologically, basidiospore size appears to separate the genera in most cases.

Most of the described species of *Hydnellum* are distributed to North America [2,27,32,33,34] and Europe [1,6,35,36], with a few species reported from Singapore, India, Australia, and New Guinea [26]. About 61 species have been described and transferred to the genus according to Index Fungorum (http://www.indexfungorum.org/ (accessed on 1 August 2021)) and MycoBank; however, only three taxa have been previously reported from China, and detailed molecular studies have not been performed [37]. Some specimens of this genus collected from China were identified as *H. aurantiacum*, *H. ferrugineum* and *H. suaveolens* based solely on morphological characteristics. However, molecular methods revealed that these specimens are misidentified, and the specimens need to be re-identified.

Numerous *Hydnellum* specimens have been collected from field investigations on stipitate hydnoid fungi in China during the past two decades. During the study of these specimens, twenty-nine new taxa have been identified using morphological characters and phylogenetic analyses of nuc rDNA ITS1-5.8S-ITS2 combined with nuc 28S rDNA, nuc 18S rDNA and nuc RPB2 rDNA sequences. In this paper, we present these taxa with illustrated morphological descriptions, phylogeny and comparison with related and/or similar taxa, a key and classification system.

The aims of this study are: (1) To describe the new taxa of *Hydnellum* from China and confirm or propose infrageneric subdivision (new subgenera, new species and newly recorded species) based on morphological and phylogenetic analyses; (2) To provide a classification system using hyphal structure types, molecularly supported clades and morphological characteristics within *Hydnellum* and *Sarcodon*; and (3) To confirm the phylogenetic position of *Hydnellum* within the Thelephorales.

## 2. Materials and Methods

### 2.1. Morphological Studies

Specimens were deposited at the herbarium of the Institute of Applied Ecology, Chinese Academy of Sciences (IFP). Microscopic procedures followed Mu et al. [38]. Structures were examined microscopically from sections mounted in Cotton Blue (CB): 0.1 mg aniline blue dissolved in 60 g pure lactic acid; CB+ = cyanophilous, CB− = acyanophilous. Amyloid and dextrinoid reactions were tested in Melzer’s reagent (IKI): 1.5 g KI (potassium iodide), 0.5 g I (crystalline iodine), 22 g chloral hydrate, 20 mL distilled water; IKI− = neither amyloid nor dextrinoid reaction. Sections were mounted in 5% KOH and studied at magnifications up to 1000× using a Nikon Eclipse E600 microscope (Tokyo, Japan) with phase contrast illumination. Dimensions were measured by the ruler in the eyepiece, with accuracy within 0.1 μm. In presenting basidiospore size ranges, 5% of the measurements at each end of the range are given in parentheses. The following abbreviations are used in the text: Lm = mean spore length, Wm = mean spore width, Q = range of length/width ratios for specimens studied, and n = total number of basidiospores measured from a given number of specimens. The surface morphology for the basidiospores was observed with a Phenom Prox scanning electron microscope (ESEM, Phenom Prox, FEI, The Netherlands) at an accelerating voltage of 20 kV. A thin layer of gold was coated on the samples to avoid charging. Special color terms are from Rayner [39] and Munsell [40].

### 2.2. Molecular Procedures and Phylogenetic Analyses

Fungal taxa and strains used in this study are listed in Table 1. Phire Plant Direct PCR Kit (Thermo Fisher Scientific, Waltham, MA, USA) procedures were used to extract total genomic DNA from the basidiocarps. Polymerase chain reactions (PCR) was performed on a Bio-Rad T100^TM^ Thermal cycler (Bio-RAD Inc., Hercules, CA, USA). Amplification reactions were performed in a 30 μL reaction mixture using the following final concentrations or total amounts: 0.9 μL template DNA, 15 μL of 2× Phire Plant PCR buffer, 1.5 μL of each primer, 0.6 μL Phire HS II DNA Polymerase, and 10.5 μL ddH_2_O (double distilled water). Primer sequences for the used genes are provided in Table 2. The PCR lthermal cycling program condition was set as follows: initial denaturation at 98 °C for 5 min, followed by 39 cycles at 98 °C for 30 s, × °C (the annealing temperatures for LROR/LR7, ITS1-F/ITS4, NS1/NS4, and bRPB2-6F/bRPB2-7.1R were 47.2 °C, 57.2 °C, 48 °C and 57.2 °C, respectively) for 30 s, 72 °C for 30 s, and a final extension at 72 °C for 1 min. PCR amplification was confirmed on 1% agarose electrophoresis gels stained with ethidium bromide [41]. DNA sequencing was performed at the Beijing Genomics Institute (BGI). All newly generated sequences were submitted to GenBank. Additional LSU rDNA, ITS rDNA, SSU rDNA and RPB2 rDNA sequences in the dataset used to establish phylogenetic relationships were downloaded from GenBank (http://www.ncbi.nlm.nih.gov/genbank/php (accessed on 10 August 2021)) and UNITE (https://unite.ut.ee/index.php (accessed on 10 August 2021)) (Table 1). Nuclear ribosomal RNA genes were used to determine the phylogenetic position of the new species. After PCR amplification, the products were sequenced in both directions and the sequences were assembled using DNAMAN 8.0. DNA sequences were aligned using MAFFT 7.110 [42]. To ensure the repeatability of the results, alignments were not manually adjusted. The best-fit evolutionary models selected by jmodeltest-2.1.10 for genes were GTR+I+G (nLSU), K80+G (ITS1), K80 (5.8S), JC+G (ITS2), TrN+I+G (SSU), K80+G (RPB2) in the first dataset (*Hydnellum* and *Sarcodon* dataset) and GTR+I+G (nLSU), K80+G (ITS1), K80+G (5.8S), K80+G (ITS2) in the second dataset (Thelephorales dataset). These models were applied in Bayesian analyses. All gaps were treated as missing data. Maximum Likelihood (ML) analysis was performed in RAxML v8.2.4 with GTR+I+G model [43]. The best tree was obtained by executing 100 rapid bootstrap inferences and thereafter a thorough search for the most likely tree using one distinct model/data partition with joint branch length optimization [44]. Bayesian analyses with MrBayes 3.2.4 [45] implementing the Markov Chain Monte Carlo (MCMC) technique and parameters predetermined with MrMODELTEST2.3 [46,47] were performed. Four simultaneous Markov chains were run starting from random trees, keeping one tree every 100th generation until the average standard deviation of split frequencies was below 0.01. The value of burn-in was set to discard 25% of trees when calculating the posterior probabilities. Bayesian posterior probabilities were obtained from the 50% majority rule consensus of the trees kept. Then the FigTree v1.3.1 were used to visualize the resulting trees.

## 3. Results

### Phylogenetic Analyses

**Figure 1:**
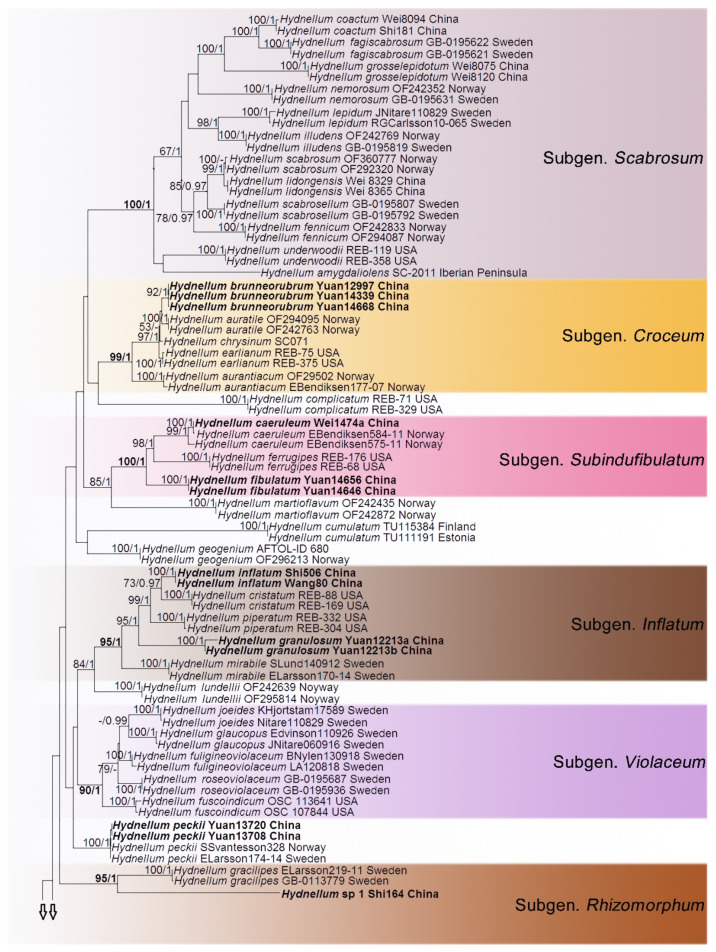

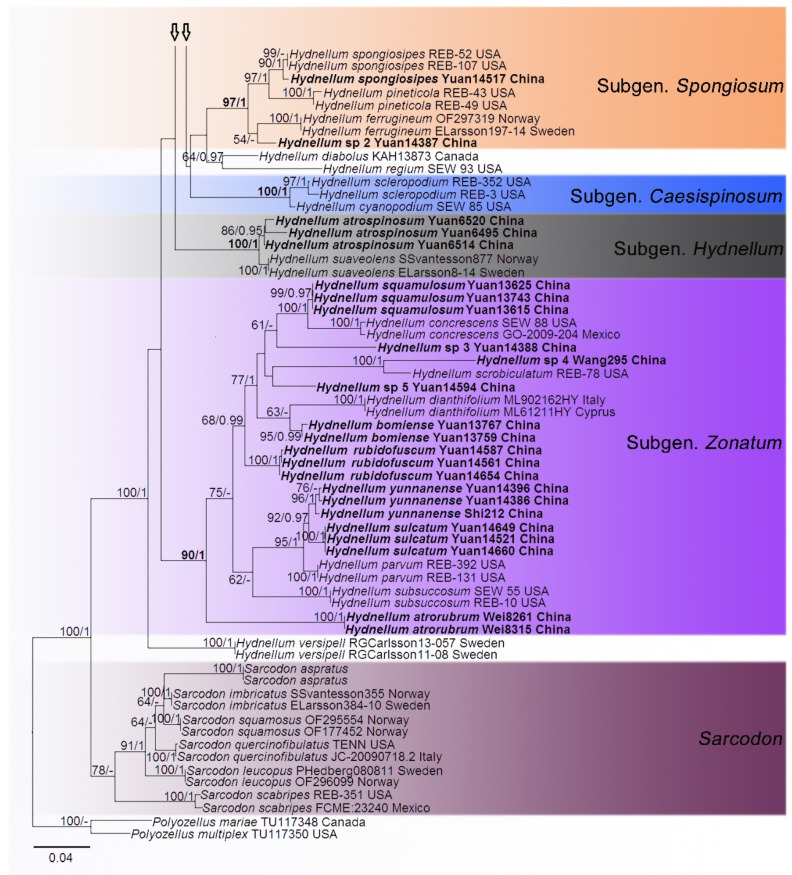
Maximum likelihood tree illustrating the phylogeny of *Hydnellum* and *Sarcodon* based on nLSU, ITS, SSU and RPB2 sequence datasets. Branches are labeled with maximum likelihood bootstrap support greater than 50 % and Bayesian posterior probabilities greater than 0.95. Newly sequenced collections are in bold.

**Figure 2:**
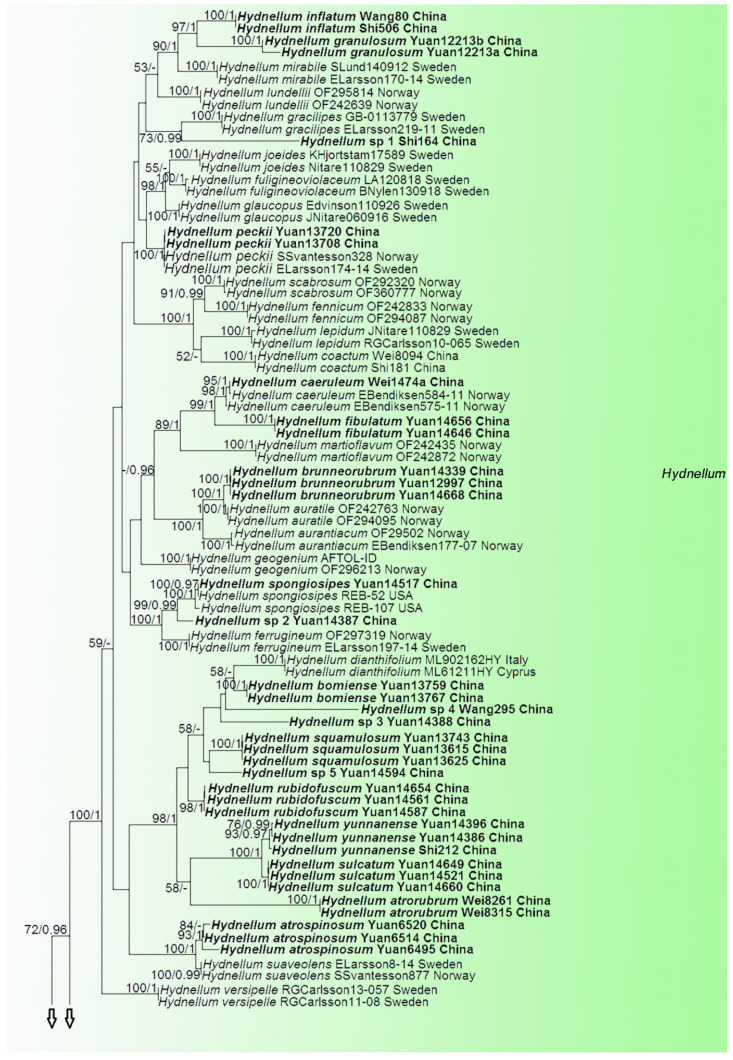

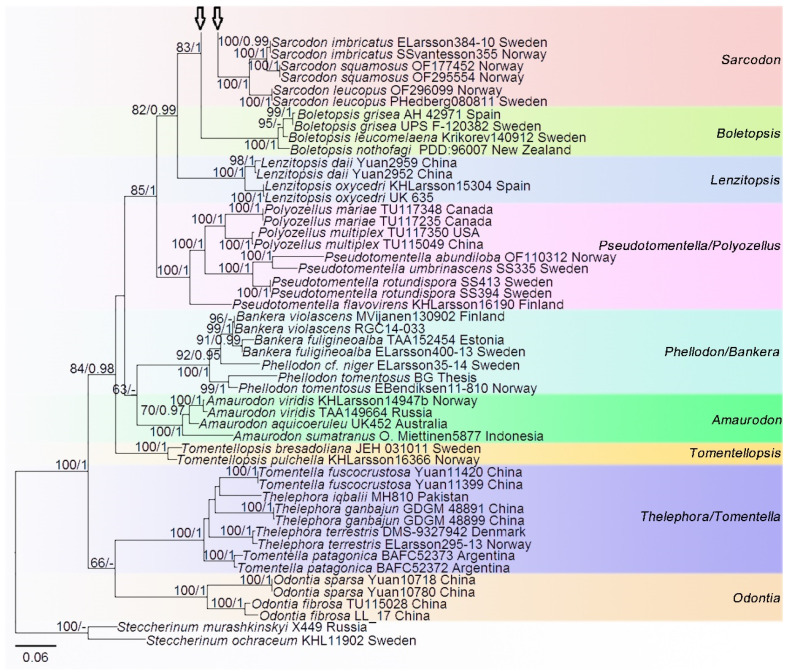
Maximum likelihood phylogenetic analysis based on the nLSU and ITS sequences of Thelephorales. Branches are labeled with maximum likelihood bootstrap support greater than 50 % and Bayesian posterior probabilities greater than 0.95. Newly sequenced collections are in bold.

In the first dataset, 272 sequences derived from four gene loci (nLSU, ITS, SSU and RPB2) were used to build phylogenetic trees; 108 of them were newly generated, including 25 of nLSU, 37 sequences of ITS, 25 of SSU and 21 of RPB2. The phylogenetic construction performed with maximum likelihood and Bayesian Inference (BI) analyses for two combined datasets showed similar topology. The combined LSU-ITS-SSU-RPB2 dataset represented 70 taxa and 3629 characters after being trimmed. *Polyozellus mariae* and *P. multiplex* were used as the outgroups according to phylogenetic analysis of Thelephorales. Bayesian analysis ran for 8 million generations and resulted in an average standard deviation of split frequencies of 0.005062. The same dataset and alignment were analysed using the ML method. The Maximum Likelihood tree is shown in Figure 1. In the phylogenetic tree, ten clades which correspond to subgenus *Hydnellum*, subg. *Caesispinosum*, subg. *Croceum*, subg. *Inflatum*, subg. *Rhizomorphum*, subg. *Scabrosum*, subg. *Spongiosum*, subg. *Subindufibulatum*, subg. *Violaceum* and subg. *Zonatum* were revealed. Twenty-eight sampled specimens formed 11 new species (*Hydnellum atrorubrum*, *H. atrospinosum*, *H. bomiense*, *H. brunneorubrum*, *H. fibulatum*, *H. granulosum*, *H. inflatum*, *H. rubidofuscum*, *H. squamulosum*, *H. sulcatum* and *H. yunnanense*) and clustered in a clade that comprised most species of *Hydnellum*. Four sampled specimens (Wei1474a, Yuan13708 and Yuan13720, Yuan14517) that were confirmed as new records from China clustered with *Hydnellum caeruleum*, *H. peckii* and *H. spongiosipes* with strong support. In addition, five notable specimens, *Hydnellum* sp 1, *Hydnellum* sp 2, *Hydnellum* sp 3, *Hydnellum* sp 4 and *Hydnellum* sp 5, formed five separate clades, and need further verification. In the second dataset, the combined ITS and nLSU gene also included sequences from 129 specimens representing 58 taxa of Thelephorales, as well as *Steccherinum ochraceum* and *S. murashkinskyi*, which were chosen as outgroups according to previous study [31]. The average standard deviation of split frequencies in the Bayesian analyses reached 0.007357 after running for 8 million generations. The calculated values based on the dataset analysed using the ML method. The Maximum Likelihood tree is shown in Figure 2. It revealed that the *Hydnellum* clade occupies an independent phylogenetic position. The *Hydnellum* clade is sister to the *Sarcodon* clade. According to the phylogenetic tree, ten major clades, *Amaurodon* clade, *Boletopsis* clade, *Hydnellum* clade, *Lenzitopsis* clade, *Odontia* clade, *Phellodon/Bankera* clade, *Pseudotomentella/Polyozellus* clade, *Sarcodon* clade, *Thelephora/Tomentella* clade and *Tomentellopsis* clade, were identified within the Thelephorales (Figure 2). Therefore, in order to use the maximum amount of genetic information when defining new species, we conducted the first dataset. Meanwhile, ITS trees of *Hydnellum* and *Sarcodon* were constructed and produced a topology similar to that generated by the first dataset (see Supplementary, Figure S1). The purpose of executing the second dataset was to demonstrate the phylogenetic position of *Hydnellum* species in the Thelephorales.

## 4. Taxonomy

***Hydnellum* subg. *Hydnellum***

MycoBank MB841191

*Etymology. Hydnellum* (Latin), refers to the subgenus in which the type species of the genus is located.

Included species: ***Hydnellum atrospinosum***, *H. suaveolens*

Type species: *Hydnellum suaveolens* (Scop.) P. Karst.

Notes: This subgenus consists of the genus type *Hydnellum suaveolens* and our new species *H. atrospinosum*; they share the characteristics of dark blue context, decurrent and dark spines, clamped generative hyphae in the context and the spines trama and irregularly oblong, tuberculate basidiospores of similar size. Furthermore, both species occur in coniferous forests [27,34].

**Figure 3:**
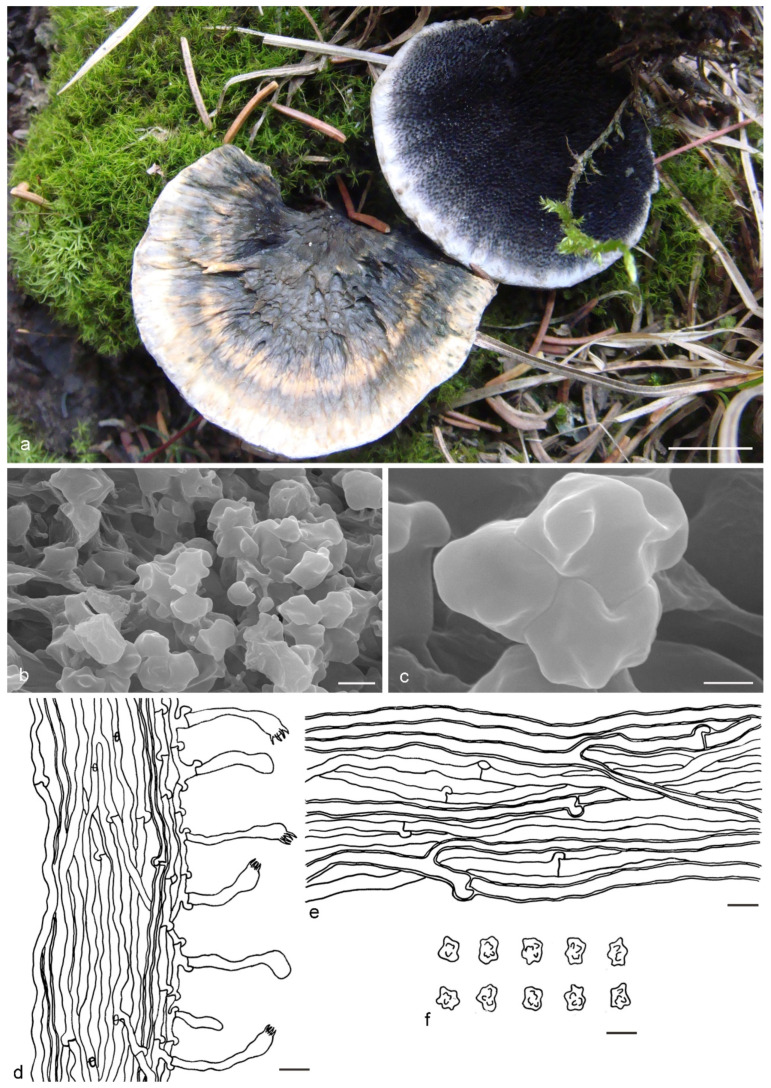
*Hydnellum atrospinosum.* (**a**): Basidiocarps; (**b**,**c**): SEM of basidiospores; (**d**–**f**): Microscopic structures (drawn from IFP 018516); (**d**): Section of hymenophore trama with basidia; (**e**): Hyphae from pileus context; (**f**): Basidiospores. —Scale bars: (**a**) = 1 cm; (**b**) = 3 μm; (**c**) = 1 μm; (**d**,**e**) = 10 µm; (**f**) = 5 µm.

***Hydnellum atrospinosum*** Y.H. Mu & H.S. Yuan, **sp. nov.** (Figure 3)

MycoBank MB839034

*Etymology*. *Atrospinosum* (Latin), refers to the dark violet spines.

*Type:* CHINA, Qinghai Province, Qilian County, Binggou Forest Park, ground in *Picea* forest, 8 September 2012, *H. S. Yuan*, Yuan 6520 (holotype IFP 018516).

*Basidiocarps* annual, solitary to gregarious or concrescent, leathery when fresh, becoming hard and light in weight upon drying; taste mild, odor fragrant when dry. *Pileus* irregularly ellipsoid to circular, later flabelliform or semicircular and applanate with age, up to 75 mm diam and 6–11 mm thick at center. *Pileal surface* light orange (5A4) to yellowish brown (5F6), concentrically zonate, scrobiculate when fresh, becoming glabrescent, rugose when dry; margin white (5A1) when fresh, brownish orange (5C6) when dry, even. *Spine surface* dark violet (15F4) when fresh, violet-gray (15F2) when dry; spines up to 2.5 mm long, base up to 0.4 mm diam, conical, 3–4 per mm, decurrent on stipe, without spines at pileus margin, brittle when dry. *Context* not duplex, up to 11 mm thick, light yellow (4A4), brownish gray (7F2) to dark violet (16F5) or dark blue (20E6), woody. *Stipe* lateral, up to 4 cm long and 3 cm diam, sometimes connate, leathery when fresh, woody upon drying, brown (6E6) to violet gray (15F2), glabrous, inside solid, cylindrical to flatted or broadened below with bulbous base when old. *Hyphal structure:* hyphal system monomitic; generative hyphae with clamp-connections, CB+ in slightly thick-walled hyphae, IKI–; tissues olivaceous in KOH. *Context:* generative hyphae hyaline, thin- to slightly thick-walled, moderately branched, clamped, straight, regularly arranged, sometimes flexuous and collapsed, mostly 3–6 μm diam. *Spines:* generative hyphae hyaline, thin- to slightly thick-walled, moderately branched, more or less parallel along spines, clamped, straight, 2–3 μm diam. *Cystidia* and cystidioles absent. *Basidia* clavate, thin-walled, with four sterigmata (2.1–4.2 μm long), clamped at base, 17–45 × 3–6 μm; basidioles similar to basidia. *Basidiospores* irregular oblong or triangular, brown, thin-walled, tuberculate CB–, IKI–, (4–)4.1–5.1(–5.5) × (3–)3.1–3.9(–4) μm, Lm = 4.6 μm, Wm = 3.2 μm, Q = 1.34–1.44 (n = 60/2); tuberculi isolated, sometimes in groups of two or more, then bi- to trifurcate in shape, up to 1 μm long.

Additional specimens (paratypes) examined: CHINA, Qinghai Province, Qilian County, Binggou Forest Park, ground in *Picea* forest, 8 September 2012, *H. S. Yuan*, Yuan 6495 (IFP 018495, paratype); Yuan 6514 (IFP 018510, paratype).

Notes: *Hydnellum atrospinosum* and *H. suaveolens* have a close phylogenetic relationship with full support (100% in ML and 1.00 BPP). Morphologically, they both have single to gregarious basidiocarps with glabrous to rugose pileal surface, woody and dark blue context, an eccentric and terete stipe with a bulbous base, conical, decurrent and dark spines, clamped generative hyphae and irregularly oblong, tuberculate basidiospores of similar size. However, *H. suaveolens* differs from *H. atrospinosum* by longer spines (up to 6 mm vs. 2.5 mm in *H. atrospinosum*), context tissues turning light blue to green in KOH and presence of inflated hyphae in the context [27,34]. A special characteristic of *H. atrospinosum* is that the clamped generative hyphae are present in all parts of the basidiocarp; this trait can also be observed in *H. cruentum*, *H. cyanopodium*, *H. geogenium* and *H. scleropodium*. *H. cruentum* differs from *H. atrospinosum* by plushy to tomentose pileal surface, grayish blue and slightly longer spines (up to 3.5 mm vs. 2.5 mm in *H. atrospinosum*) and subglobose basidiospores [26,27,51]. *H. cyanopodium* and *H. scleropodium* obviously differs in blue spines [27,33,51]. *H. geogenium* differs in reflexed-multiplex and yellow basidiocarps, pale yellow to brown spines and subglobose basidiospores [1,27,34].

***Hydnellum* subg. *Caesispinosum*** Y.H. Mu & H.S. Yuan, **subgen. nov.**

MycoBank MB841195

*Etymology. Caesispinosum* (Latin), refers to the blue spines.

Included species: *Hydnellum cyanopodium, H. scleropodium*

Type species: *Hydnellum cyanopodium* K.A. Harrison

Notes: The subgenus is composed of two American species, *Hydnellum cyanopodium* and *H. scleropodium*. Blue spines and context and fully clamped hyphae in all parts of basidiocarps are their distinctly common features. In addition, the two species both have rugose and pitted pileal surface and clamped basidia of similar size [33,34,51].

***Hydnellum* subg. *Croceum*** Y.H. Mu & H.S. Yuan, **subgen. nov.**

MycoBank MB841196

*Etymology. Croceum* (Latin), refers to the orange basidiocarps.

Included species: *Hydnellum aurantiacum*, *H. auratile*, ***H. brunneorubrum***, *H. chrysinum*, *H. earlianum*

Type species: *Hydnellum aurantiacum* (Batsch) P. Karst.

Notes: This subgenus includes five species, *Hydnellum aurantiacum*, *H. auratile*, *H. brunneorubrum*, *H. chrysinum* and *H. earlianum*. They often have orange basidiocarps, tomentose to matted pileal surface, yellow to orange spines and context, the monomitic hyphal system with uninflated and unclamped hyphae, clavate basidia with simple-septate at base and irregularly subglobose basidiospores [2,26,27,33,34,51].

**Figure 4:**
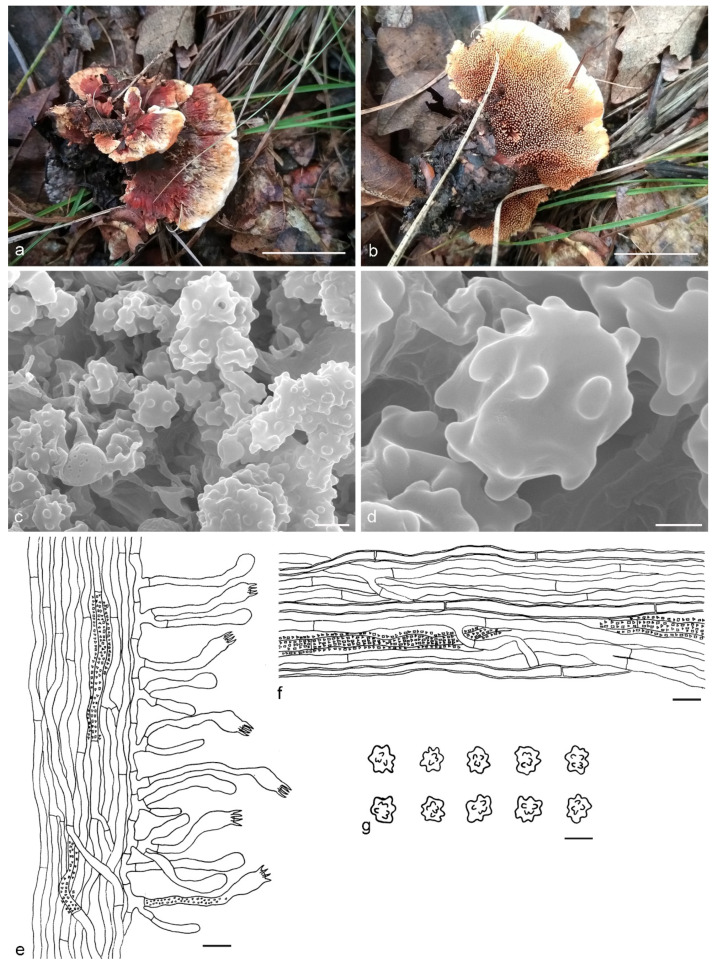
*Hydnellum brunneorubrum.* (**a**,**b**): Basidiocarps; (**c**,**d**): SEM of basidiospores; (**e**–**g**): Microscopic structures (drawn from IFP 019384); (**e**): Section of hymenophore trama with basidia; (**f**): Hyphae from pileus context; (**g**): Basidiospores. —Scale bars: (**a**,**b**) = 1 cm; (**c**) = 3 μm; (**d**) = 1 μm; (**e**,**f**) = 10 µm; (**g**) = 5 µm.

***Hydnellum brunneorubrum*** Y.H. Mu & H.S. Yuan, **sp. nov.** (Figure 4)

MycoBank MB839036

Type: CHINA, Liaoning Province, Xinbin County, Gangshan Nature Reserve, on the ground in Fagaceous forest, 30 August 2018, *H. S. Yuan*, Yuan 12997 (holotype IFP 019384).

*Basidiocarps* annual, solitary to gregarious or multiple pilei overlapping and fused to form a compound cluster, soft and leathery when fresh, becoming woody and light in weight upon drying; taste mild, odor none when dry. *Pileus* applanate and ellipsoid to irregularly circular when young, later depressed or infundibuliform to flabelliform with age, up to 40 mm diam and 5–10 mm thick at center. *Pileal surface* brownish orange (6C8) to brownish red (10D8), azonate, pubscent to floccose when fresh, becoming matted or fibrillose to glabrous when dry; margin white (6A1) to light orange (6A5) when fresh, light orange (5A4) when dry, involute and wavy, sometimes lobed with age. *Spine surface* golden yellow (5B6) to light brown (7D8) when fresh, light brown (6D7) to dark brown (7F8) when dry; spines up to 4 mm long, base up to 0.3 mm diam, conical, 3–5 per mm, more or less decurrent on stipe, without spines at pileus margin, brittle when dry. *Context* not duplex, up to 5 mm thick, grayish orange (5B5), woody. *Stipe* central to lateral, up to 3 cm long and 1 cm diam, sometimes connate, leathery when fresh, woody upon drying, brownish orange (6C8) to light brown (6D7), tomentose, solid inner, cylindrical to flat or attenuate downwards with bulbous base when old. *Hyphal structure:* hyphal system monomitic; generative hyphae with simple-septa, occasionally encrusted, CB+ in slightly thick-walled hyphae, IKI–; tissues olivaceous in KOH. *Context:* generative hyphae hyaline, thin- to slightly thick-walled, frequently branched, simple-septate, straight, regularly arranged, sometimes flexuous and collapsed, mostly 4–6 μm diam. *Spines:* generative hyphae hyaline, thin-walled, frequently branched, more or less parallel along spines, frequently simple-septate, straight, 2–5 μm diam. *Cystidia* and cystidioles absent. *Basidia* clavate, thin-walled, with four sterigmata (2.5–5 μm long), simple-septate at base, 12–50 × 3–7 μm; basidioles similar to basidia. *Basidiospores* irregularly ellipsoid to subglobose, brown, thin-walled, tuberculate, CB–, IKI–, (4–)4.1–5.1(–5.2) × (3.1–)3.2–4.6(–4.8) μm, Lm = 4.9 μm, Wm = 3.9 μm, Q = 1.23–1.26 (n = 60/2); tuberculi usually isolated, sometimes in groups of two or more, then bi- to trifurcate in shape, up to 0.8 μm long.

Additional specimens (paratypes) examined: CHINA, Liaoning Province, Xinbin County, Gangshan Nature Reserve, on the ground in mixed forest, 30 August 2018, *H. S. Yuan*, Yuan 12999 (IFP 019385, paratype); Yuan 13004 (IFP 019386, paratype); 2 September 2019, *H. S. Yuan*, Yuan 14339 (IFP 019387, paratype); Yuan 14340 (IFP 019388, paratype); Yuan 14341 (IFP 019389, paratype); 12 August 2020, *H. S. Yuan*, Yuan 14562 (IFP 019390, paratype); 26 August 2020, *H. S. Yuan*, Yuan 14585 (IFP 019391, paratype); 12 September 2020, *H. S. Yuan*, Yuan 14796 (IFP 019392, paratype); Yuan 14798 (IFP 019393, paratype); Yuan 14799 (IFP 019394, paratype); Benxi County, Guanmenshan National Forest Park, on the ground in mixed forest, 29 August 2020, *H. S. Yuan*, Yuan 14642 (IFP 019395, paratype); Xinbin County, Qingsongling Forest Park, on the ground in mixed forest, 5 September 2020, *H. S. Yuan*, Yuan 14668 (IFP 019396, paratype); Yuan 14688 (IFP 019397, paratype).

Notes: The orange basidiocarps make *Hydnellum brunneorubrum* similar to *H. aurantiacum*, *H. auratile*, *H. chrysinum* and *H. earlianum*. *H. aurantiacum* differs from *H. brunneorubrum* in bigger pileus (up to 100 mm vs. 40 mm in *H. brunneorubrum*) with colliculose and wrinkled pileal surface, longer spines (up to 5 mm vs. up to 4 mm in *H. brunneorubrum*) and longer basidiospores (6–6.7 μm vs. 4.1–5.1 μm in *H. brunneorubrum*) [1]. *H. auratile* differs in squamulose, concentrically zoned and occasionally black stained pileal surface, duplex stipe context with black lines in the centre and longer basidiospores (4.9–5.8 μm vs. 4.1–5.1 μm in *H. brunneorubrum*); in addition, *H. auratile* usually grows in coniferous forests [1,26]. *H. chrysinum* is differentiated by having duplex, slightly zonate context, context tissue turning dark olive or blackish in KOH and slightly longer basidia sterigmata (up to 6 µm vs. up to 5 µm in *H. brunneorubrum*) [2,33,51]. *H. earlianum* is differentiated by a larger pileus (up to 90 mm vs. 40 mm in *H. brunneorubrum*), duplex context, dark brown or black context tissues in KOH and longer basidiospores (5–6 µm vs. 4.1–5.1 μm in *H. brunneorubrum*) [27,32,34].

***Hydnellum* subg. *Inflatum*** Y.H. Mu & H.S. Yuan, **subgen. nov.**

MycoBank MB841197

*Etymology. Inflatum* (Latin), refers to the presence of inflated generative hyphae.

Included species: *Hydnellum cristatum*, ***H. granulosum***, ***H. inflatum***, *H. mirabile*, *H. piperatum*

Type species: *Hydnellum mirabile* (Fr.) P. Karst.

Notes: There are five species in the subgenus *Inflatum*, *Hydnellum cristatum*, *H. granulosum*, *H. inflatum*, *H. mirabile* and *H. piperatum*. The presence of inflated generative hyphae in the context of the pileus is an important feature they share. As well as, they often have yellow to brown and depressed pileus, cylindrical stipe and unclamped generative hyphae in the context and the spine trama [1,27,34].

**Figure 5:**
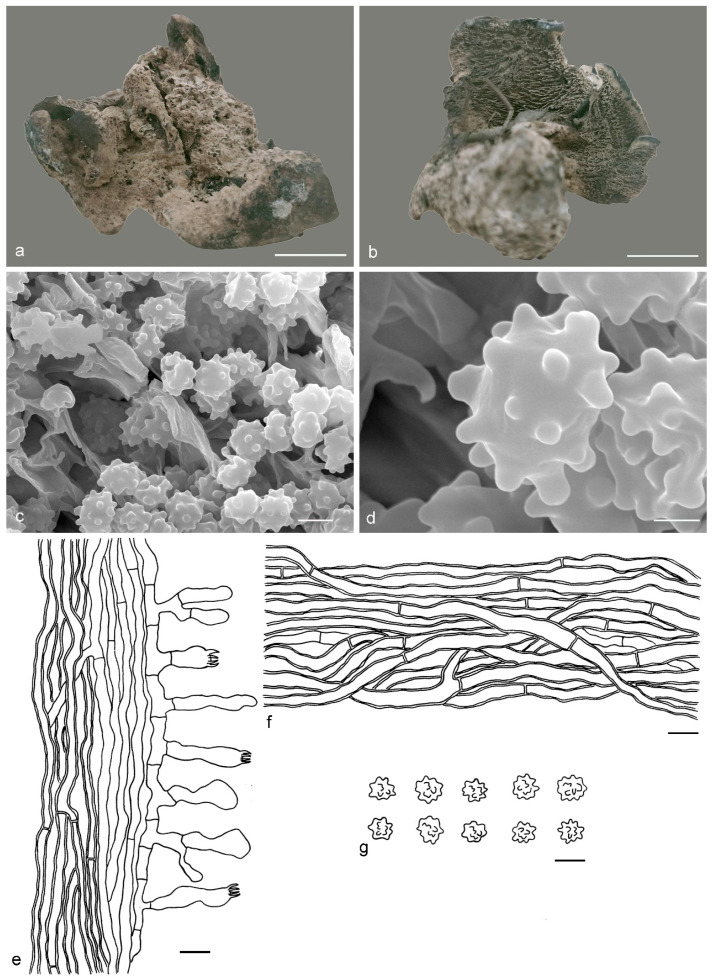
*Hydnellum granulosum.* (**a**,**b**): Basidiocarps; (**c**,**d**): SEM of basidiospores; (**e**–**g**): Microscopic structures (drawn from IFP 019400); (**e**): Section of hymenophore trama with basidia; (**f**): Hyphae from pileus context; (**g**): Basidiospores. —Scale bars: (**a**,**b**) = 1 cm; (**c**) = 3 μm; (**d**) = 1 μm; (**e**,**f**) = 10 µm; (**g**) = 5 µm.

***Hydnellum granulosum*** Y.H. Mu & H.S. Yuan, **sp. nov.** (Figure 5)

MycoBank MB839038

*Etymology*. *Granulosum* (Latin), refers to the granulose pileal surface when dry.

Type: CHINA, Sichuan Province, Guangyuan County, Tianzhaoshan National Forest Park, on the ground in *Acer* and *Cryptomeria* mixed forest, 13 August 2017, *H. S. Yuan*, Yuan 12213b (holotype IFP 019400).

*Basidiocarps* annual, solitary to gregarious or two to three pilei fused to form a complex pileus, soft and leathery when fresh, becoming hard and light in weight upon drying; taste acrid, odor fragrant when dry. *Pileus* aplanate, irregularly ellipsoid when young, later irregularly flabelliform with age, up to 50 mm diam and 5–10 mm thick at center. *Pileal surface* light yellow (4A4), light brown to grayish brown (9F3), azonate, granulose when dry; margin yellowish white (4A2), involute and wavy, sometimes lobed. *Spine surface* grayish orange (5B4) to dark brown (8F7) when dry; spines up to 2 mm long, base up to 0.3 mm diam, conical, 3–6 per mm, more or less decurrent on stipe, without spines at pileus margin, brittle when dry. *Context* not duplex, up to 9 mm thick, grayish orange (5B4), woody. *Stipe* central to lateral, up to 4 cm long and 3 cm diam, sometimes connate, hard upon drying, golden brown (5D6) to brown (6E6), rugose, solid inner, terete or attenuate or broadening downwards with bulbous base when old. *Hyphal structure:* hyphal system monomitic; generative hyphae with simple-septa, CB+ in slightly thick-walled hyphae, IKI–; tissues olivaceous in KOH. *Context:* generative hyphae hyaline, mostly slightly thick-walled, rarely thin-walled, occasionally branched, simple-septate, occasionally inflated, interwoven, mostly 4–7 μm diam. *Spines:* generative hyphae hyaline, thin- to slightly thick-walled, moderately branched, more or less parallel along spines, frequently simple-septate, straight, 3–4 μm diam. *Cystidia* and cystidioles absent. *Basidia* clavate, thin-walled, with four sterigmata (3–5 μm long), simple-septate at base, 15–30 × 5–9 μm; basidioles similar to basidia. *Basidiospores* irregularly ellipsoid to globose, brown, thin-walled, tuberculate, CB–, IKI–, (4–)4.1–5.1(–5.3) × (3.2–)3.4–4.7(–4.9) μm, Lm = 4.6 μm, Wm = 4.1 μm, Q = 1.12–1.13 (n = 60/2); tuberculi usually isolated, sometimes in groups of two or more, then bi- to trifurcate in shape, up to 1.2 μm long.

Additional specimens (paratypes) examined: CHINA, Sichuan Province, Guangyuan County, Tianzhaoshan National Forest Park, on the ground in *Acer* and *Cryptomeria* mixed forest, 12 August 2017, *H. S. Yuan*, Yuan 12213a (IFP 019401, paratype); 13 August 2017, *H. S. Yuan*, Yuan 12213c (IFP 019402, paratype).

Notes: *Hydnellum granulosum* has a close phylogenetic relationship with *H. piperatum.* In morphology, *H. piperatum* resembles *H. granulosum* in having single to gregarious or concrescent basidiocarps, with lobed pileal margin from fused pilei or indeterminate growth, a single to fused, central to eccentric and terete to attenuate downwards stipe, context tissue turning olivaceous in KOH, absence of clamp-connections and the presence of inflated hyphae. However, *H. piperatum* is distinguishable from *H. granulosum* by having a comparatively broader pileus (up to 150 mm vs. 50 mm in *H. granulosum*) with a scaly or squamulose and zonate pileal surface and strongly decurrent and longer spines (up to 5 mm vs. 2 mm in *H. granulosum*) [27]. The presence of inflated generative hyphae is a shared feature in *H. granulosum* and *H. mirabile*. They also have simple or concrescent basidiocarps with light yellow to brown pilei, and hard and cylindrical stipes. However, *H. mirabile* differs from *H. granulosum* by the plano-convex to depressed and larger pileus (up to 90 mm vs. 50 mm in *H. granulosum*), yellowish to purplish brown and longer spines (up to 5 mm vs. 2 mm in *H. granulosum*), duplex and pallid to pale brownish context and longer basidiospores (5.6–5.8 µm vs. 4.1–5.1 μm in *H. granulosum*) [1].

**Figure 6:**
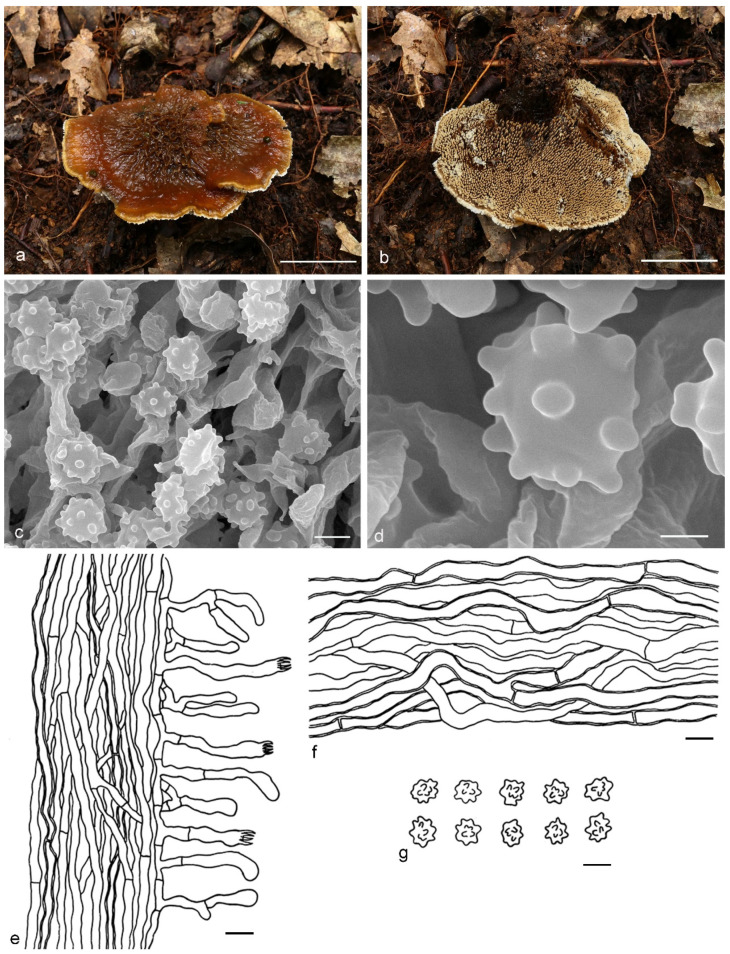
*Hydnellum inflatum.* (**a**,**b**): Basidiocarps; (**c**,**d**): SEM of basidiospores; (**e**–**g**): Microscopic structures (drawn from IFP 019403); (**e**): Section of hymenophore trama with basidia; (**f**): Hyphae from pileus context; (**g**): Basidiospores. —Scale bars: (**a**,**b**) = 1 cm; (**c**) = 3 μm; (**d**) = 1 μm; (**e**,**f**) = 10 µm; (**g**) = 5 µm.

***Hydnellum inflatum*** Y.H. Mu, X.H. Wang & H.S. Yuan, **sp. nov.** (Figure 6)

MycoBank MB839040

*Etymology*. *Inflatum* (Latin), refers to the mostly inflated generative hyphae in the context of the pileus.

Type: CHINA, Yunnan Province, Maguan County, Dalishu Township, on the ground in Fagaceous forest, 14 October 2017, *S. F. Shi*, Shi 506 (holotype IFP 019403).

*Basidiocarps* annual, solitary to gregarious, soft and leathery when fresh, becoming corky and light in weight upon drying; taste acrid, odor fragrant when dry. *Pileus* depressed to aplanate and irregularly circular when young, later flabelliform with age, up to 75 mm diam and 3–8 mm thick at center. *Pileal surface* grayish orange (5B5) to brown (7E7), azonate, fibrous to colliculose when fresh, becoming scrobiculate when dry; margin light yellow (4A4) when fresh, orange-white (5A2) when dry, involute and wavy, often lobed with age. *Spine surface* white (5A1) to golden brown (5D7) when fresh, yellowish brown (5D5) to dark brown (7F8) when dry; spines up to 4 mm long, base up to 0.25 mm diam, conical, 4–5 per mm, strongly decurrent on stipe, without spines at pileus margin, brittle when dry. *Context* duplex, upper layer grayish yellow (4B5), loose and soft, up to 2 mm thick; lower layer pale yellow (4A3), woody, up to 3 mm thick; a dark line present between upper and lower cortical layers. *Stipe* central to lateral, up to 5 cm long and 2 cm diam, occasionally connate, fleshy when fresh, woody upon drying, light brown (6D7), smooth to rugose, inside solid, context with a dark line present at centre, cylindrical or attenuate downwards with bulbous base when old. *Hyphal structure:* hyphal system monomitic; generative hyphae with simple-septa, CB+ in slightly thick-walled hyphae, IKI–; tissues slightly olivaceous in KOH. *Context:* generative hyphae hyaline, thin- to slightly thick-walled, occasionally branched, simple-septate, mostly inflated, interwoven, mostly 4–8 μm diam. *Spines:* generative hyphae hyaline, thin- to slightly thick-walled, moderately branched, more or less parallel along spines, frequently simple-septate, straight, 2–5 μm diam. *Cystidia* and cystidioles absent. *Basidia* clavate, thin-walled, with four sterigmata (3–5 μm long), simple-septate at base, 19–33 × 4–6 μm; basidioles similar to basidia. *Basidiospores* irregularly ellipsoid to globose, brown, thin-walled, tuberculate, CB–, IKI–, (4–)4.2–5(–5.1) × (3.2–)3.8–4.3(–5) μm, Lm = 4.8 μm, Wm = 4 μm, Q = 1.18–1.2 (n = 60/2); tuberculi usually isolated, sometimes in groups of two or more, then bi- to trifurcate in shape, up to 1.2 μm long.

Additional specimens (paratypes) examined: CHINA, Yunnan Province, Maguan County, Mabai Town, Caiyuanzi Village, on the ground, 5 August 2017, *J. Wang*, Wang 80 (IFP 019404, paratype); Wang 82 (IFP 019405, paratype); on the way from Dalishu Township to Damagu Village, on the ground in Fagaceous forest, 6 August 2017, *S. F. Shi*, Shi 150 (IFP 019406, paratype); Shi 160 (IFP 019407, paratype).

Notes: *Hydnellum inflatum* is characterized by the presence of inflated generative hyphae, which makes it similar to *H. cristatum*, *H. granulosum*, *H. mirabile* and *H. piperatum*. This is an important feature that distinguishes from other species. However, *H. cristatum* can be differentiated from *H. inflatum* by the larger pileus (up to 100 mm vs. 75 mm in *H. inflatum*) with tomentose to matted pileal surface, longer spines (up to 5 mm vs. 4 mm in *H. inflatum*), brown context tissues in KOH, bigger basidia (34–46 × 8–9 μm vs. 19–33 × 4–6 μm in *H. inflatum*) and basidiospores (5–6 × 4–5 μm vs. 4.2–5 × 3.8–4.3 μm in *H. inflatum*) [27,34]. *H. granulosum* differs in granulose pileal surface, not duplex context, mostly slightly thick-walled hyphae in the context and shorter spines (up to 2 mm vs. up to 4 mm in *H. inflatum*). *H. mirabile* differs in ochraceous yellow and olive brown pileus, yellowish to purplish brown spines and longer spores (5.6–5.8 μm vs. 4.2–5 μm in *H. inflatum*) [1]. *H. piperatum* differs in umbilicate and greatly pileus (up to 100 mm vs. 75 mm in *H. inflatum*), red haired to sunburn slightly longer spines (up to 5 mm vs. up to 4 mm in *H. inflatum*) and inflated to cylindrical hyphae in the spines [27].

***Hydnellum* subg. *Rhizomorphum*** Y.H. Mu & H.S. Yuan, **subgen. nov.**

MycoBank MB841198

*Etymology. Rhizomorphum* (Latin), refers to the rhizomorphs-like stipe.

Included species: *Hydnellum gracilipes*, ***Hydnellum* sp 1**

Type species: *Hydnellum gracilipes* (P. Karst.) P. Karst.

Notes: *Hydnellum gracilipes* and our notable specimen *Hydnellum* sp 1 comprise the subgenus *Rhizomorphum*. Rhizomorph-like stipe are their typical common characteristics. Besides, both species have the monomitic hyphal system with simple-septate generative hyphae [35].

***Hydnellum* sp 1**

*Basidiocarps* annual, solitary to gregarious or coalescent. *Pileus* flabelliform to irregularly circular. *Pileal surface* deep red (10C8) to violet-brown (10F8) and felted. *Spines* white (10A1) to brownish red (10D7) and more or less decurrent, up to 1.5 mm long. *Rhizomorphs* stipe-like. *Hyphal system* monomitic, generative hyphae simple-septa. *Basidia* clavate, with four sterigmata. *Basidiospores* irregularly subglobose to globose, tuberculate, (4–)4.1–4.9(–5) × (3.1–)3.2–4(–4.1) μm.

Specimen examined: CHINA, Yunnan Province, Maguan County, on the way from Dalishu Township to Damagu Village, on the ground of angiosperm forest, 6 August 2017, *S. F. Shi*, Shi 164 (IFP 019436).

***Hydnellum* subg. *Scabrosum*** Y.H. Mu & H.S. Yuan, **subgen. nov.**

MycoBank MB841199

*Etymology. Scabrosum* (Latin), refers to the pileal surface with scabrosity.

Included species: *Hydnellum amygdaliolens*, *H. coactum*, *H. fagiscabrosum*, *H. fennicum*, *H. grosselepidotum*, *H. illudens*, *H. lepidum*, *H. lidongensis*, *H. nemorosum*, *H. scabrosellum*, *H. scabrosum*, *H. underwoodii*

Type species: *Hydnellum scabrosum* (Fr.) E. Larss., K.H. Larss. & Kõljalg

Notes: There are twelve species, namely *Hydnellum amygdaliolens*, *H. coactum*, *H. fagiscabrosum*, *H. fennicum*, *H. grosselepidotum*, *H. illudens*, *H. lepidum*, *H. lidongensis*, *H. nemorosum*, *H. scabrosellum*, *H. scabrosum* and *H. underwoodii* in this subgenus characterised by planar to depressed and brown pileus, azonate pileal surface with scabrosity, variously-brown spines, not duplex and yellow to orange context, inflated and unclamped generative hyphae and irregularly ellipsoid to globose basidiospore [1,2,26,27,32,33,34,38,51,52,53,54].

***Hydnellum* subg. *Spongiosum*** Y.H. Mu & H.S. Yuan, **subgen. nov.**

MycoBank MB841200

*Etymology. Spongiosum* (Latin), refers to the spongy pileal surface.

Included species: *Hydnellum ferrugineum*, *H. pineticola*, ***H. spongiosipes***, ***Hydnellum*** **sp 2**

Type species: *Hydnellum ferrugineum* (Fr.) P. Karst.

Notes: This subgenus includes *Hydnellum ferrugineum*, *H. pineticola*, *H. spongiosipes* and *Hydnellum* sp 2; they all have planoconvex to depressed and brown pileus with spongy pileal surface, purplish brown spines, the monomitic hyphal system with simple-septate generative hyphae and irregularly subglobose basidiospores [1,2,27,34].

***Hydnellum spongiosipes*** (Peck) Pouzar, Česká Mykol. 14(2): 130 (1960)

*Hydnum spongiosipes* Peck, Ann. Rep. Reg. N.Y. St. Mus. 50: 111 (1898) (1897)

*Basidiocarps* single to gregarious. *Pileus* flabelliform, applanate to subdepressed. *Pileal surface* pale orange (6A3) to dark brown (6F7), velutinous to very spongy, tomentose to fibrillose. *Spines* pale orange (6A3) to dark brown (6F7), subdecurrent to decurrent, up to 6 mm long. *Stipe* central to subeccentric, terete, thick and strong. *Hyphal system* monomitic; generative hyphae simple-septate. *Basidia* clavate, with four sterigmata. *Basidiospores* irregularly subglobose, tuberculate, (5–)5.1–6.1(–6.2) × (4.3–)4.5–5.3(–5.8) μm.

Specimen examined: CHINA, Liaoning Province, Kuandian County, Baishilazi National Nature Reserve, on the ground of *Quercus* forest, 8 August 2020, *H. S. Yuan*, Yuan 14517 (IFP 019435).

Notes: The studied sample clustered with *Hydnellum spongiosipes* (REB-107 and REB-52) in the multi-gene phylogenetic tree with strong support (90% in ML and 1.00 BPP) (Figure 1). The samples REB-107 and REB-52 both show 0.99 similarity to Yuan 14517 in ITS region. Besides this, morphological analyses also confirmed the new record, which is described in detail by Maas Geesteranus (1975) and Baird (2013). This species was recorded to occur widely in the United States and European countries and usually was found under hardwood tree [1,2,27,34].

***Hydnellum* sp 2**

*Basidiocarps* annual, coalescent. *Pileus* compound, multiple pilei fused. *Pileal surface* violet-brown (10E6) and spongy-tomentose. *Spines* white (10A1) to violet-brown (10E6) and strongly decurrent, up to 2.5 mm long. *Stipe* short and connate. *Hyphal system* monomitic, generative hyphae simple-septa. *Basidia* clavate, with four sterigmata. *Basidiospores* irregularly subglobose, tuberculate, (3.1–)4–4.3(–4.5) × (3–)3.1–4(–4.1) μm.

Specimen examined: CHINA, Yunnan Province, Nanjian County, Lingbaoshan National Forest Park, on the ground of angiosperm forest, 19 September 2019, *H. S. Yuan*, Yuan 14387 (IFP 019437).

***Hydnellum* subg. *Subindufibulatum*** Y.H. Mu & H.S. Yuan, **subgen. nov.**

MycoBank MB841201

*Etymology. Subindufibulatum* (Latin), refers to the occasionally clamped hyphae in the context of the pileus.

Included species: ***Hydnellum caeruleum***, *H. ferrugipes*, ***H. fibulatum***

Type species: *Hydnellum caeruleum* (Hornem.) P. Karst.

Notes: *Hydnellum caeruleum*, *H. ferrugipes* and *H. fibulatum* make up the subgenus *Subindufibulatum* and occasional presence of clamped hyphae in the context of the pileus is the dominating trait that distinguishes them from other species. Furthermore, they have dark brown and fibrillose to colliculose pileal surface, orange stipe, context tissues turning olivaceous in KOH and the absence of clamp-connections in the spine trama [2,26,27].

***Hydnellum caeruleum*** (Hornem.) P. Karst., [as ‘*coeruleum*’], Meddn Soc. Fauna Flora Fenn. 5: 41 (1879)

*Hydnum caeruleum* Hornem., Fl. Danic. 8(23): 7, tab. 1374 (1808)

*Basidiocarps* single to gregarious or concrescent. *Pileus* convex to plane. *Pileal surface* pastel yellow (4A4) to dark blonde (5D4), tomentose and colliculose. *Spines* decurrent, up to 6 mm long, orange-white (5A2) to dark brown (6F8). *Stipe* central and terete. *Hyphal system* monomitic; most of the generative hyphae with simple-septa, rarely with clamps. *Basidia* clavate, with four sterigmata. *Basidiospores* irregularly subglobose, tuberculate, (4.9–)5–6(–6.1) × (4–)4.1–4.9(–5) μm.

Specimen examined: CHINA, Xinjiang Autonomous Region, Huocheng County, Guozigou Forest Park, on the ground in *Picea* forest, 18 August 2004, *Y. L. Wei*, Wei 1474a (IFP 019432).

Notes: The phylogenetic analyses showed that the studied sample matched with *Hydnellum caeruleum* (EBendiksen584-11) with full support (100% in ML and 1.00 BPP) (Figure 1). ITS sequence BLAST also revealed it is 100% identical to *H. caeruleum*. Besides, our collection shares identical characters with *H. caeruleum* described by Maas Geesteranus [26] in morphology. This is the first report of this species from China.

**Figure 7:**
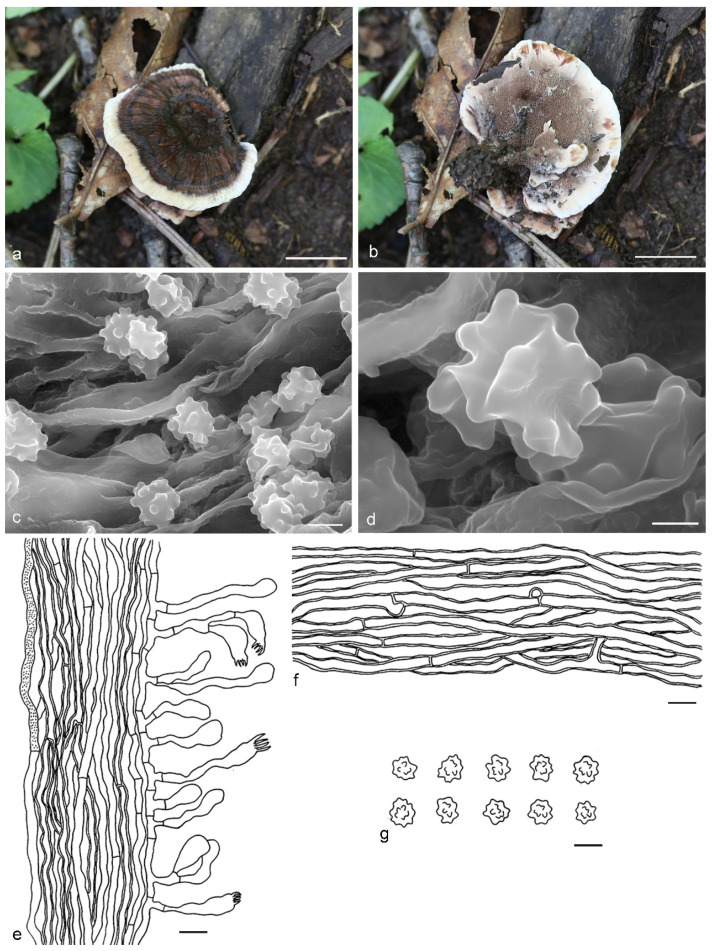
*Hydnellum fibulatum.* (**a**,**b**): Basidiocarps; (**c**,**d**): SEM of basidiospores; (**e**–**g**): Microscopic structures (drawn from IFP 019398); (**e**): Section of hymenophore trama with basidia; (**f**): Hyphae from pileus context; (**g**): Basidiospores. —Scale bars: (**a**,**b**) = 2 cm; (**c**) = 3 μm; (**d**) = 1 μm; (**e**,**f**) = 10 µm; (**g**) = 5 µm.

***Hydnellum fibulatum*** Y.H. Mu & H.S. Yuan, **sp. nov.** (Figure 7)

MycoBank MB839037

*Etymology*. *Fibulatum* (Latin), refers to the generative hyphae with occasional clamp-connections.

Type: CHINA, Liaoning Province, Benxi County, Guanmenshan National Forest Park, on the ground in *Quercus* forest, 29 August 2020, *H. S. Yuan*, Yuan 14656 (holotype IFP 019398).

*Basidiocarps* annual, solitary to gregarious, soft and leathery when fresh, becoming woody and light in weight upon drying; taste mild, odor none when dry. *Pileus* applanate, circular when young, later flabelliform with age, up to 45 mm diam and 3–7 mm thick at center. *Pileal surface* light brown (7D7) to dark brown (8F4), obscurely zonate, pubescent when fresh, becoming fibrillose, rugose when dry; margin white (6A1) when fresh, brown (6E6) when dry, incurved, sometimes lobed. *Spine surface* pinkish white (7A2) to brown (7E7) when fresh, orange-white (5A2) to brown (6E6) when dry; spines up to 1.5 mm long, base up to 0.2 mm diam, conical, 3–4 per mm, more or less decurrent on stipe, without spines at pileus margin, brittle when dry. *Context* not duplex, up to 5 mm thick, light brown (6D4) to dark brown (6F4), woody. *Stipe* central to lateral, up to 3.5 cm long and 1 cm diam, single, leathery when fresh, woody upon drying, light orange (5A5) to brown (6E7), pubescent, solid inner, cylindrical or attenuate downwards with bulbous base when old. *Hyphal structure:* hyphal system monomitic; generative hyphae with mostly simple-septa, occasionally clamped, CB+ in slightly thick-walled hyphae, IKI–; tissues olivaceous in KOH. *Context:* generative hyphae hyaline, slightly thick-walled, occasionally branched, simple-septate, occasionally clamped, straight, regularly arranged, sometimes flexuous and collapsed, mostly 3–5 μm diam. *Spines:* generative hyphae hyaline, thin- to slightly thick-walled, moderately branched, more or less parallel along spines, frequently simple-septate, straight, 2–4 μm diam. *Cystidia* and cystidioles absent. *Basidia* clavate, thin-walled, with four sterigmata (2–5 μm long), simple-septate at base, 15–47 × 5–9 μm; basidioles similar to basidia. *Basidiospores* irregularly ellipsoid to globose, brown, thin-walled, tuberculate, CB–, IKI–, (4.2–)4.4–5.8(–6) × (4–)4.1–4.9(–5.1) μm, Lm = 5.2 μm, Wm = 4.3 μm, Q = 1.12–1.21 (n = 60/2); tuberculi usually isolated, sometimes in groups of two or more, then bi- to trifurcate in shape, up to 1 μm long.

Additional specimen (paratype) examined: CHINA, Liaoning Province, Benxi County, Guanmenshan National Forest Park, on the ground in *Quercus* forest, 29 August 2020, *H. S. Yuan*, Yuan 14646 (IFP 019399, paratype).

Notes: *Hydnellum caeruleum* and *H. ferrugipes* have an adjacent phylogenetic relationship with *H. fibulatum* according to the phylogenetic tree (Figure 1). *H. caeruleum* and *H. fibulatum* have similar morphological characteristics, such as a flat and velutinous pileus when immature, white pileal margin when fresh, central, terete and tomentose stipe, olivaceous context tissue in KOH, presence of occasional clamp-connections in the context and simple-septate hyphae in the spines. However, *H. caeruleum* can be distinguished by having a larger pileus (up to 80 mm vs. 45 mm in *H. fibulatum*), rough or colliculose pileal surface when mature, duplex and zonate context [2,26]. *H. ferrugipes* resembles *H. fibulatum* in having a white pileal margin when fresh, tomentose and orange to brown stipe, orange-white to brown spines when dry, regularly arranged and occasionally clamped hyphae in the context, unclamped hyphae in the spines and basidia sterigmata with similar size. However, *H. ferrugipes* differs from *H. fibulatum* in the infundibuliform pileus with pitted to subnodulose or subcolliculose pileal surface, blue-gray or grayish orange context, considerably longer spines (up to 6 mm vs. 1.5 mm in *H. fibulatum*) and wider basidiospores (5–6 μm vs. 4.1–4.9 μm in *H. fibulatum*) [27,34].

***Hydnellum*subg.*Violaceum*** Y.H. Mu & H.S. Yuan, **subgen. nov.**

MycoBank MB841202

*Etymology. Violaceum* (Latin), refers to the violaceous basidiocarps.

Included species: *Hydnellum fuligineoviolaceum*, *H. fuscoindicum*, *H. glaucopus*, *H. joeides*, *H. roseoviolaceum*

Type species: *Hydnellum fuligineoviolaceum* (Kalchbr.) E. Larss., K.H. Larss. & Kõljalg

Notes: Five species, *Hydnellum fuligineoviolaceum*, *H. fuscoindicum*, *H. glaucopus*, *H. joeides* and *H. roseoviolaceum*, comprise the subgenus *Violaceum*. They share the following features: violaceous basidiocarps, pileal surface with appressed scales, purplish context, the presence of inflated generative hyphae and the simple-septate haphae in all tissue [1,26,27,33,53].

***Hydnellum*subg.*Zonatum*** Y.H. Mu & H.S. Yuan, **subgen. nov.**

MycoBank MB841203

*Etymology. Zonatum* (Latin), refers to the concentrically zonate pileal surface.

Included species: ***Hydnellum atrorubrum***, ***H. bomiense***, *H. concrescens*, *H. dianthifolium*, *H. parvum*, ***H. rubidofuscum***, *H. scrobiculatum*, ***H. squamulosum***, *H. subsuccosum*, ***H. sulcatum***, ***H. yunnanense***, **Hydnellum** **sp 3**, *Hydnellum* **sp 4**, ***Hydnellum*** **sp 5**

Type species: *Hydnellum scrobiculatum* (Fr.) P. Karst. 

Notes: The subgenus *Zonatum* contains fourteen taxa, *Hydnellum atrorubrum*, *H. bomiense*, *H. concrescens*, *H. dianthifolium*, *H. parvum*, *H. rubidofuscum*, *H. scrobiculatum*, *H. squamulosum*, *H. subsuccosum*, *H. sulcatum*, *H. yunnanense*, *Hydnellum* sp 3*, Hydnellum* sp 4 and *Hydnellum* sp 5. The concentrically zonate pileal surface is their most prominently mutual peculiarity. Additionally, the absence of clamp-connections in the context of the pileus and the spine trama is another important common feature [1,2,26,27,34,36].

**Figure 8:**
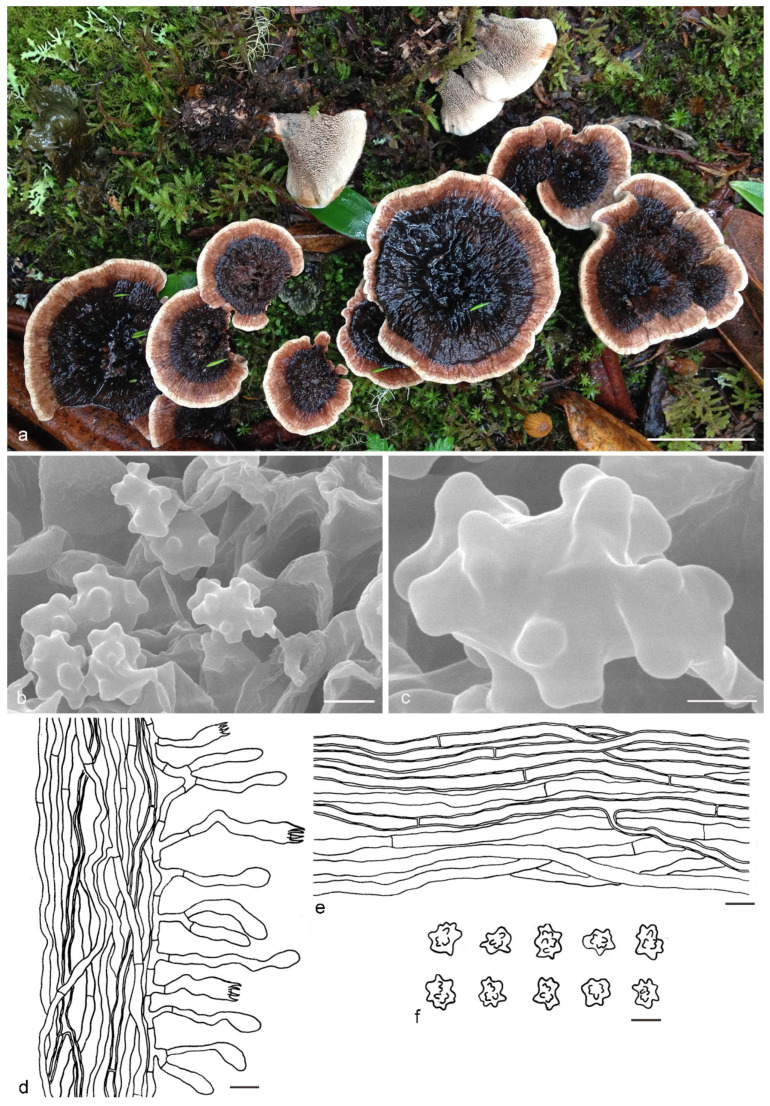
*Hydnellum atrorubrum.* (**a**): Basidiocarps; (**b**,**c**): SEM of basidiospores; (**d**–**f**): Microscopic structures (drawn from IFP 019377); (**d**): Section of hymenophore trama with basidia; (**e**): Hyphae from pileus context; (**f**): Basidiospores. —Scale bars: (**a**) = 2.5 cm; (**b**) = 3 μm; (**c**) = 1 μm; (**d**,**e**) = 10 µm; (**f**) = 5 µm.

***Hydnellum atrorubrum*** Y.H. Mu & H.S. Yuan, **sp. nov.** (Figure 8)

MycoBank MB839032

*Etymology*. *Atrorubrum* (Latin), refers to the dark ruby red pileal surface.

Type: CHINA, Yunnan Province, Yulong County, on the ground in Fagaceous forest, 23 July 2018, *Y. L. Wei*, Wei 8261 (holotype IFP 019377).

*Basidiocarps* annual, solitary to gregarious or several pilei fused to form a compound pileus, soft and leathery when fresh, becoming woody and light in weight upon drying; taste slightly bitter, odor slightly fragrant when dry. *Pileus* applanate and flabelliform to irregularly circular when young, later depressed or subinfundibuliform to rounded with age, up to 48 mm diam and 3–8 mm thick at center. *Pileal surface* light brown (7D7) to dark ruby (12F8), usually concentrically zoned, flocculose when fresh, becoming fibrillose to glabrescent when dry; margin white (7A1) when fresh, brown (7E5) when dry, even to slightly irregular, occasionally wavy or lobed. *Spine surface* white (6A1) to dark brown (6F6) when fresh, grayish orange (5B4) to brown (6E8) when dry; spines up to 3.5 mm long, base up to 0.4 mm diam, conical, 3–5 per mm, decurrent to strongly decurrent on stipe, without spines at pileus margin, brittle when dry. *Context* not duplex, up to 6 mm thick, brown (6E6), corky. *Stipe* central, up to 3.5 cm long and 1.5 cm diam, sometimes connate, leathery when fresh, corky upon drying, pinkish (8A2) to reddish brown (8E6), velutinous, inside solid, cylindrical or attenuate downwards with bulbous base when old. *Hyphal structure:* hyphal system monomitic; generative hyphae with simple-septa, CB+ in slightly thick-walled hyphae, IKI–; tissues olivaceous in KOH. *Context:* generative hyphae hyaline, thin- to slightly thick-walled, occasionally branched, simple-septate, straight, regularly arranged, sometimes flexuous and collapsed, mostly 4–6 μm diam. *Spines:* generative hyphae hyaline, thin- to slightly thick-walled, moderately branched, more or less parallel along spines, frequently simple-septate, straight, 2–4 μm diam. *Cystidia* and cystidioles absent. *Basidia* clavate, thin-walled, with four sterigmata (4–6 μm long), simple-septate at base, 20–48 × 5–8 μm; basidioles similar to basidia. *Basidiospores* irregular ellipsoid, brown, thin-walled, tuberculate, CB–, IKI–, (4.1–)4.5–6(–6.1) × (3.2–)3.9–5.1(–6) μm, Lm = 5 μm, Wm = 4.4 μm, Q = 1.14–1.21 (n = 60/2); tuberculi usually isolated, sometimes in groups of two or more, then bi- to trifurcate in shape, up to 1.1 μm long.

Additional specimens (paratypes) examined: CHINA, Yunnan Province, Yulong County, on the ground in Fagaceous forest, *Y. L. Wei*, 23 July 2018, Wei 8290 (IFP 019378, paratype); Wei 8312 (IFP 019379, paratype); Wei 8315 (IFP 019380, paratype); Wei 8319 (IFP 019381, paratype).

Notes: Phylogenetically, *Hydnellum atrorubrum* has a close relationship with *H. subsuccosum*. Morphologically, *H. subsuccosum* is similar to *H. atrorubrum* in having gregarious to confluent basidiocarps with zonate pileal surface and lobed pileal margin, brown context, cylindrical or attenuate downwards stipe, decurrent and similar length spines, the monomitic hyphal system with uninflated and simple-septate generative hyphae, basidia of similar shape and width, as well as basidiospores of similar length. However, *H. subsuccosum* can be differentiated by scabrous to nodulose and orange-white to camel pileal surface, black spines and presence of subglobose basidiospores [27]. *H. auratile* is comparable to *H. atrorubrum* in having similar size, depressed to infundibuliform or flabelliform and concentrically zoned pileus with undulate margin, tomentose and reddish-brown stipe, non-duplex context, thin- to slightly thick-walled and unclamped generative hyphae. However, *H. auratile* differs from *H. atrorubrum* by orange or orange-brown to dark red-brown pileus with entire or deeply split margin and tawny to purplish brown spines [26].

**Figure 9:**
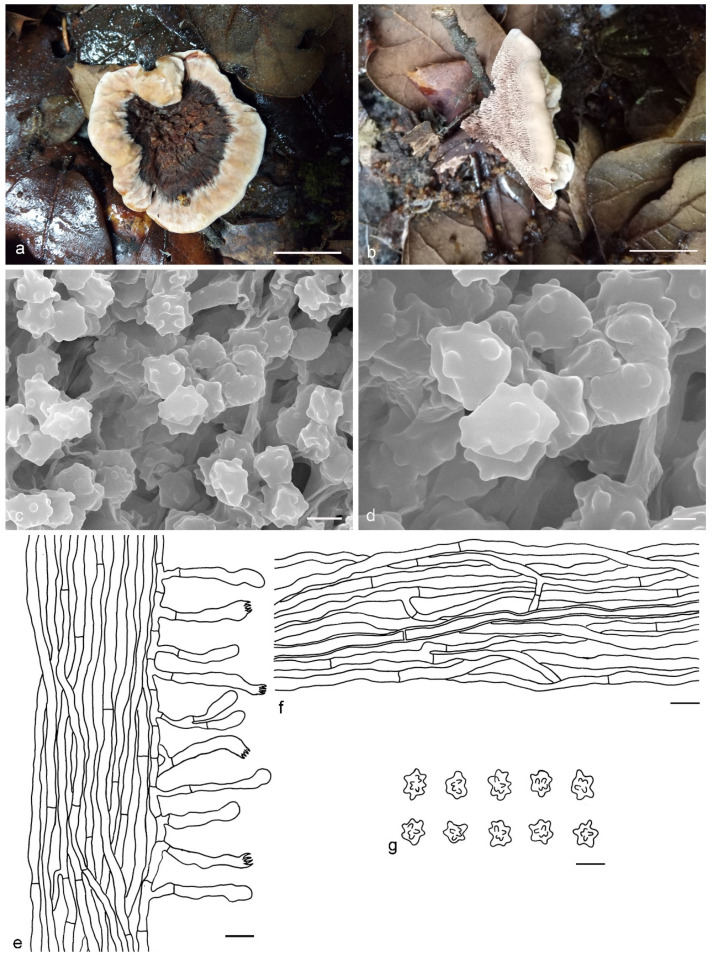
*Hydnellum bomiense.* (**a**,**b**): Basidiocarps; (**c**,**d**): SEM of basidiospores; (**e**–**g**): Microscopic structures (drawn from IFP 019382); (**e**): Section of hymenophore trama with basidia; (**f**): Hyphae from pileus context; (**g**): Basidiospores. —Scale bars: (**a**,**b**) = 1 cm; (**c**) = 3 μm; (**d**) = 1 μm; (**e**,**f**) = 10 µm; (**g**) = 5 µm.

***Hydnellum bomiense*** Y.H. Mu & H.S. Yuan, **sp. nov.** (Figure 9)

MycoBank MB839035

*Etymology*. *Bomiense*, refers to the Bomi County, where the specimens were collected.

Type: CHINA, Xizang Autonomous Region, Bomi County, on the ground in Fagaceous forest, 19 July 2019, *H. S. Yuan*, Yuan 13767 (holotype IFP 019382).

*Basidiocarps* annual, solitary to gregarious, soft and leathery when fresh, becoming woody and light in weight upon drying; taste acrid, odor slightly fragrant when dry. *Pileus* infundibuliform when young, later applanate and irregularly circular with age, up to 26 mm diam and 2–4 mm thick at center. *Pileal surface* grayish yellow (4B4), brown (7E7) to dark brown (7F8), obscurely concentrically zonate, tomentose, scrupose when fresh, becoming fibrillose or glabrous when dry; margin white (5A1) when fresh, grayish orange (5B4) when dry, involute and wavy, sometimes lobed or rimose with age. *Spine surface* white (6A1) to brown (6E7) when fresh, light brown (6D6) to dark brown (7F8) when dry; spines up to 1.1 mm long, base up to 0.2 mm diam, conical, 4–6 per mm, more or less decurrent on stipe, without spines at pileus margin, brittle when dry. *Context* not duplex, up to 4 mm thick, brown (6E5), woody. *Stipe* central to lateral, up to 2 cm long and 0.5 cm diam, woody upon drying, grayish orange (5B5) to dark brown (7F7), rugose, solid inner, terete or attenuate downwards with bulbous base when old. *Hyphal structure:* hyphal system monomitic; generative hyphae with simple-septa, CB+ in slightly thick-walled hyphae, IKI–; tissues olivaceous in KOH. *Context:* generative hyphae hyaline, thin- to slightly thick-walled, frequently branched, simple-septate, straight, regularly arranged, sometimes flexuous and collapsed, mostly 4–6 μm diam. *Spines:* generative hyphae hyaline, thin-walled, occasionally branched, more or less parallel along spines, frequently simple-septate, straight, 2–4 μm diam. *Cystidia* and cystidioles absent. *Basidia* clavate, thin-walled, with four sterigmata (1.5–3 μm long), simple-septate at base, 15–42 × 4–7 μm; basidioles similar to basidia. *Basidiospores* irregularly ellipsoid to subglobose, brown, thin-walled, tuberculate, CB–, IKI–, (4–)4.1–5.1(–5.2) × (3–)3.3–4.5(–4.8) μm, Lm = 4.7 μm, Wm = 4 μm, Q = 1.18–1.21 (n = 60/2); tuberculi usually isolated, sometimes in groups of two or more, then bi- to trifurcate in shape, up to 1 μm long.

Additional specimen (paratype) examined: CHINA, Xizang Autonomous Region, Bomi County, on the ground with moss in Fagaceous forest, 19 July 2019, *H. S. Yuan*, Yuan 13759 (IFP 019383, paratype).

Notes: This species clustered with two samples from Estonia and Costa Rica, and formed an independent clade (Figure S1). *Hydnellum bomiense* and *H. dianthifolium* are closely related based on nucleotide sequence analyses and possess common morphological features: separate to coalescing or grouped basidiocarps with decurrent spines, cylindrical stipe, absence of clamp-connections and brown, tuberculate and irregularly ellipsoid to subglobose basidiospores of similar size with isolated to bifurcate tuberculi. However, *H. dianthifolium* differs from *H. bomiense* by having slender, turbinate and coralloid basidiocarps that split radially to form erect, coralloid or flower-shaped lobed pilei, not perceptibly zoned context and thick-walled and encrusted context hyphae [36]. Meanwhile, *H. concrescens* and *H. scrobiculatum* are also in a big clade with *H. bomiense* in Figure 1, and concentrically zonate pileal surface is their common characteristic. However, *H. concrescens* differs by quite larger pileus (up to 120 mm vs. up to 26 mm in *H. bomiense*), duplex context and longer basidia sterigmata (3–4 µm vs. 1.5–3 µm in *H. bomiense*) [27,34]. *H. scrobiculatum* differs in subcolliculose to scrobiculate pileal surface and longer spines (up to 3 mm vs. 1.1 mm in *H. bomiense*) [27,34].

**Figure 10:**
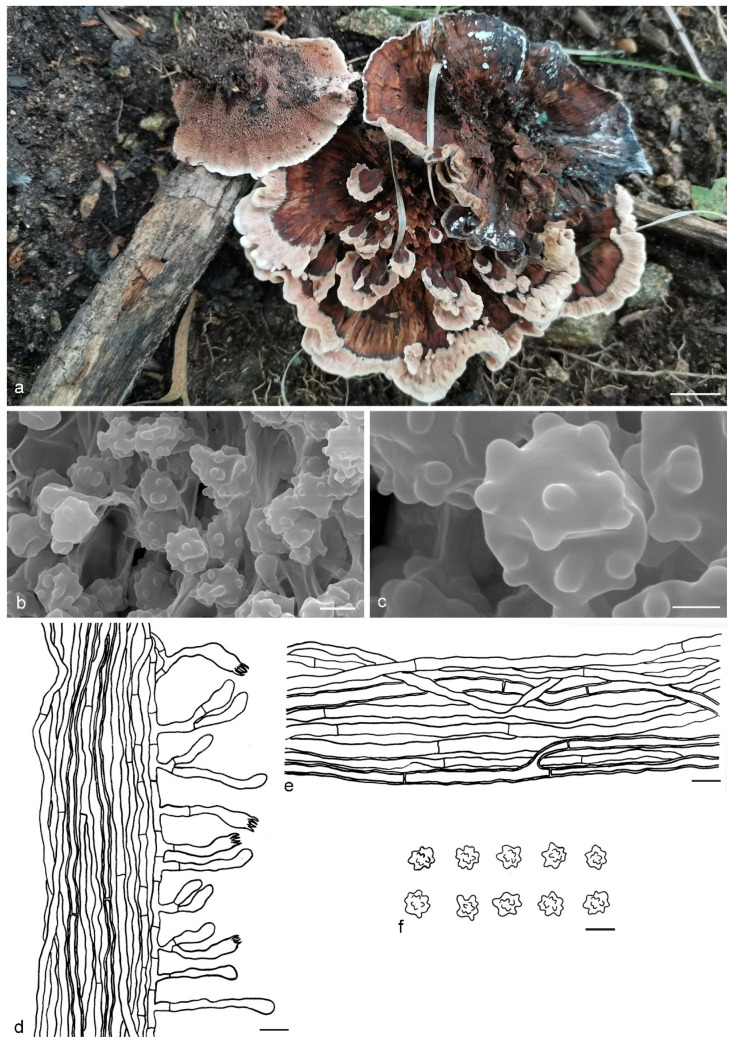
*Hydnellum rubidofuscum.* (**a**): Basidiocarps; (**b**,**c**): SEM of basidiospores; (**d**–**f**): Microscopic structures (drawn from IFP 019408); (**d**): Section of hymenophore trama with basidia; (**e**): Hyphae from pileus context; (**f**): Basidiospores. —Scale bars: (**a**) = 1 cm; (**b**) = 3 μm; (**c**) = 1 μm; (**d**,**e**) = 10 µm; (**f**) = 5 µm.

***Hydnellum rubidofuscum*** Y.H. Mu & H.S. Yuan, **sp. nov.** (Figure 10) 

MycoBank MB839041

*Etymology*. *Rubidofuscum* (Latin), refers to the reddish brown pileal surface.

Type: CHINA, Liaoning Province, Xinbin County, Gangshan Nature Reserve, on the ground in *Quercus* forest, 12 August 2020, *H. S. Yuan*, Yuan 14561 (holotype IFP 019408).

*Basidiocarps* annual, solitary to gregarious or multiple pilei overlapping and fused to form a compound cluster, soft and leathery when fresh, becoming woody and light in weight upon drying; taste mild, odor slightly fragrant when dry. *Pileus* applanate to infundibuliform when young, later depressed to flabelliform or irregularly circular with age, up to 70 mm diam and 4–10 mm thick at center. *Pileal surface* reddish brown (8E8), obscurely concentrically zonate, glabrous to scrupose when fresh, becoming fibrillose to virgate when dry; margin white (6A1) to orange-white (6A2) when fresh, brownish orange (6C4) when dry, even, sometimes lobed with age. *Spine surface* grayish brown (8D3) to reddish brown (8E7) when fresh, brown (6E6) to dark brown (6F7) when dry; spines up to 3 mm long, base up to 0.2 mm diam, conical, 4–6 per mm, strongly decurrent on stipe, without spines at pileus margin, brittle when dry. *Context* not duplex, up to 10 mm thick, brown (6E6), woody. *Stipe* central to lateral, up to 3.5 cm long and 2 cm diam, sometimes connate, leathery when fresh, woody upon drying, light brown (7D6) to brown (7E6), pubescent, solid inner, cylindrical to flat or attenuate downwards with bulbous base when old. *Hyphal structure:* hyphal system monomitic; generative hyphae with simple-septa, CB+ in slightly thick-walled hyphae, IKI–; tissues olivaceous in KOH. *Context:* generative hyphae hyaline, thin- to slightly thick-walled, occasionally branched, simple-septate, straight, regularly arranged, sometimes flexuous and collapsed, mostly 3–6 μm diam. *Spines:* generative hyphae hyaline, thin-walled, occasionally branched, more or less parallel along spines, frequently simple-septate, straight, 2–3 μm diam. *Cystidia* and cystidioles absent. *Basidia* clavate, thin-walled, with four sterigmata (1–4 μm long), simple-septate at base, 14–37 × 4–6 μm; basidioles similar to basidia. *Basidiospores* irregularly ellipsoid to subglobose, brown, thin-walled, tuberculate, CB–, IKI–, (4–)4.1–5(–5.1) × (3.8–)3.9–4.6(–4.8) μm, Lm = 4.6 μm, Wm = 4.1 μm, Q = 1.11–1.12 (n = 60/2); tuberculi usually isolated, sometimes in groups of two or more, then bi- to trifurcate in shape, up to 1 μm long.

Additional specimens (paratypes) examined: CHINA, Liaoning Province, Xinbin County, Gangshan Nature Reserve, on the ground in *Quercus* forest, 12 August 2020, *H. S. Yuan*, Yuan 14559 (IFP 019409, paratype); Yuan 14560 (IFP 019410, paratype); Yuan 14563 (IFP 019411, paratype); 26 August 2020, *H. S. Yuan*, Yuan 14586 (IFP 019412, paratype); Yuan 14587 (IFP 019413, paratype); 12 September 2020, *H. S. Yuan*, Yuan 14792 (IFP 019414, paratype); Yuan 14794 (IFP 019415, paratype); Yuan 14800 (IFP 019416, paratype); Benxi County, Guanmenshan National Forest Park, on the ground in *Quercus* forest, 29 August 2020, *H. S. Yuan*, Yuan 14654 (IFP 019417, paratype).

Notes: Phylogenetically, *Hydnellum rubidofuscum* is closely related to *H. bomiense* and *H. dianthifolium* (Figure 1). Morphologically, infundibuliform pileus when young, concentrically zonate pileal surface and irregularly ellipsoid to subglobose basidiospores are their common features. However, *H. bomiense* can be distinguished by smaller pileus (up to 26 mm vs. up to 70 mm in *H. rubidofuscum*), yellow to dark brown pileal surface and shorter spines (up to 1.1 mm vs. up to 3 mm in *H. rubidofuscum*). *H. dianthifolium* differs by subpubescent pileal surface, reddish-brown to vinaceous-brown context, thick-walled and rarely branched hyphae in the context and context tissue turning blue-green in KOH [36]. The reddish brown pileal surface is very similar to that of *H. scrobiculatum*. Furthermore, *H. scrobiculatum* also has a single to gregarious or concrescent basidiocarp with applanate to depressed or infundibuliform pileus, obscurely concentrically zonate pileal surface, simple or connate, central to eccentric and velutinous stipe, reddish brown and decurrent spines and the monomitic hyphal system. However, the major differences are that *H. scrobiculatum* has a fungoid or no odor, a scrobiculate and rugulose pileal surface, duplex and zonate context and longer basidiospores (5.4–6.4 μm vs. 4.1–5 μm in *H. rubidofuscum*) [26,27,34].

**Figure 11:**
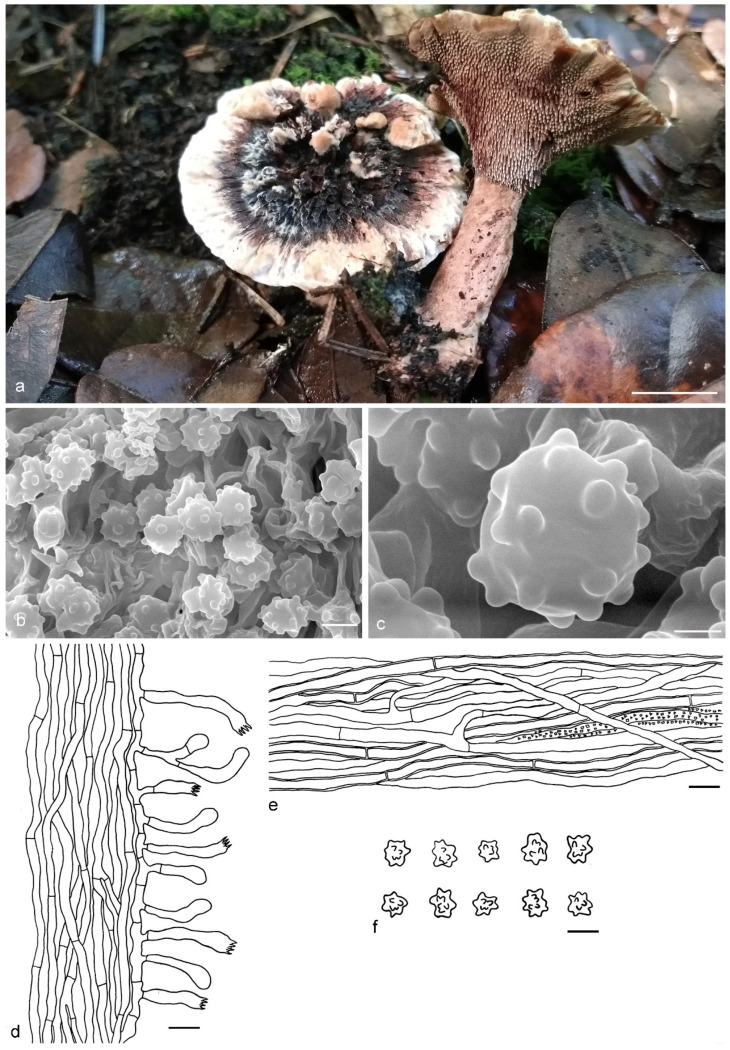
*Hydnellum squamulosum.* (**a**): Basidiocarps; (**b**,**c**): SEM of basidiospores; (**d**–**f**): Microscopic structures (drawn from IFP 019418); (**d**): Section of hymenophore trama with basidia; (**e**): Hyphae from pileus context; (**f**): Basidiospores. —Scale bars: (**a**) = 1 cm; (**b**) = 3 μm; (**c**) = 1 μm; (**d**,**e**) = 10 µm; (**f**) = 5 µm.

***Hydnellum squamulosum*** Y.H. Mu & H.S. Yuan, **sp. nov.** (Figure 11)

MycoBank MB839042

*Etymology*. *Squamulosum* (Latin) refers to the minutely scaly pileal surface.

*Holotype*. CHINA, Xizang Autonomous Region, Bomi County, on the ground in *Picea* mixed forest, 17 July 2019, *H. S. Yuan*, Yuan 13615 (holotype IFP 019418).

*Basidiocarps* annual, solitary to gregarious or coalescent to form complex pileus, soft and leathery when fresh, becoming corky and light in weight upon drying; taste none, odor none when dry. *Pileus* circular when young, circular or semicircular with age, applanate, up to 35 mm diam and 4–8 mm thick at center. *Pileal surface* pastel red (7A4) to dark Magenta (13F7), zonate, floccose to woolly, squamulose when fresh, becoming fibrillose and scrobiculate when dry; margin white (7A1) when fresh, grayish orange (5B3) when dry, involute and wavy, sometimes lobed with age. *Spine surface* pale red (7A3) to reddish brown (8E8) when fresh, grayish orange (5B4) to dark brown (8F7) when dry; spines up to 2 mm long, base up to 0.2 mm diam, conical, 3–6 per mm, decurrent on stipe, without spines at pileus margin, brittle when dry. *Context* not duplex, up to 9 mm thick, reddish brown (8E6), soft corky. *Stipe* central to lateral, up to 4 cm long and 1 cm diam, sometimes connate, leathery or freshy when fresh, soft corky upon drying, pale red (11A3), tomentose, solid inner, context with a dark zone present at centre, terete to flat or attenuate downwards with bulbous base when old. *Hyphal structure:* hyphal system monomitic; generative hyphae with simple-septa, occasionally encrusted, CB+ in slightly thick-walled hyphae, IKI–; tissues olivaceous in KOH. *Context:* generative hyphae hyaline, thin- to slightly thick-walled, moderately branched, simple-septate, straight, regularly arranged, sometimes flexuous and collapsed, mostly 4–5 μm diam. *Spines:* generative hyphae hyaline, thin-walled, moderately branched, more or less parallel along spines, frequently simple-septate, straight, 2–4 μm diam. *Cystidia* and cystidioles absent. *Basidia* clavate, thin-walled, with four sterigmata (2–3 μm long), simple-septate at base, 8–38 × 4–6 μm; basidioles similar to basidia. *Basidiospores* irregularly ellipsoid to globose, brown, thin-walled, tuberculate, CB–, IKI–, (4–)4.1–5(–5.1) × (3.2–)3.3–4.1(–4.2) μm, Lm = 4.4 μm, Wm = 3.8 μm, Q = 1.14–1.16 (n = 60/2); tuberculi usually isolated, sometimes in groups of two or more, then bi- to trifurcate in shape, up to 1 μm long.

Additional specimens (paratypes) examined: CHINA, Xizang Autonomous Region, Bomi County, on the ground in *Picea* mixed forest, 17 July 2019, *H. S. Yuan*, Yuan 13617 (IFP 019419, paratype); Yuan 13625 (IFP 019420, paratype); Yuan 13626 (IFP 019421, paratype); Yuan 13627 (IFP 019422, paratype); on the ground with moss in *Picea* mixed forest, 19 July 2019, *H. S. Yuan*, Yuan 13743 (IFP 019423, paratype).

Notes: *Hydnellum squamulosum* and *H. concrescens* are closely related in the phylogenetic tree and share similar morphological and anatomical characteristics: a solitary to gregarious or coalescent basidiocarp with fibrillose, squamulose and zonate pileal surface, irregularly lobed margin, decurrent and reddish-brown spines, not duplex context in the pileus, zonate context in the stipe, context tissue becoming olivaceous in KOH, and tuberculate basidiospores. However, *H. concrescens* can be differentiated by depressed or infundibuliform basidiocarps, reddish white to dark brown pileal margin, larger pileus (up to 120 mm vs. 35 mm in *H. squamulosum*), longer basidia sterigmata (up to 5 µm vs. up to 3 µm in *H. squamulosum*) and larger basidiospores (5–6 × 4–5 µm vs. 4.1–5 × 3.3–4.1 μm in *squamulosum*) [2,27,34]. *H. fraudulentum* is similar to *H. squamulosum* in having a squamulose-fibrillose pileal surface, cylindrical or connate stipe, context tissue olivaceous in KOH, thin- to slightly thick-walled and unclamped hyphae in the context, basidia of similar shape, and brown and tuberculate basidiospores. However, it differs from *H. squamulosum* in having depressed, azonate and yellow-brown to dark brown pilei, purplish brown and slightly longer spines (up to 2.5 mm vs. 2 mm in *H. squamulosum*), wider basidia (6–7 µm vs. 4–6 μm in *H. squamulosum*) with longer sterigmata (3.6–4.5 µm vs. 2–3 μm in *H. squamulosum*) and bigger basidiospores (6.3–7 × 4.5–4.7 µm vs. 4.1–5 ×3.3–4.1 μm in *H. squamulosum*) [26].

**Figure 12:**
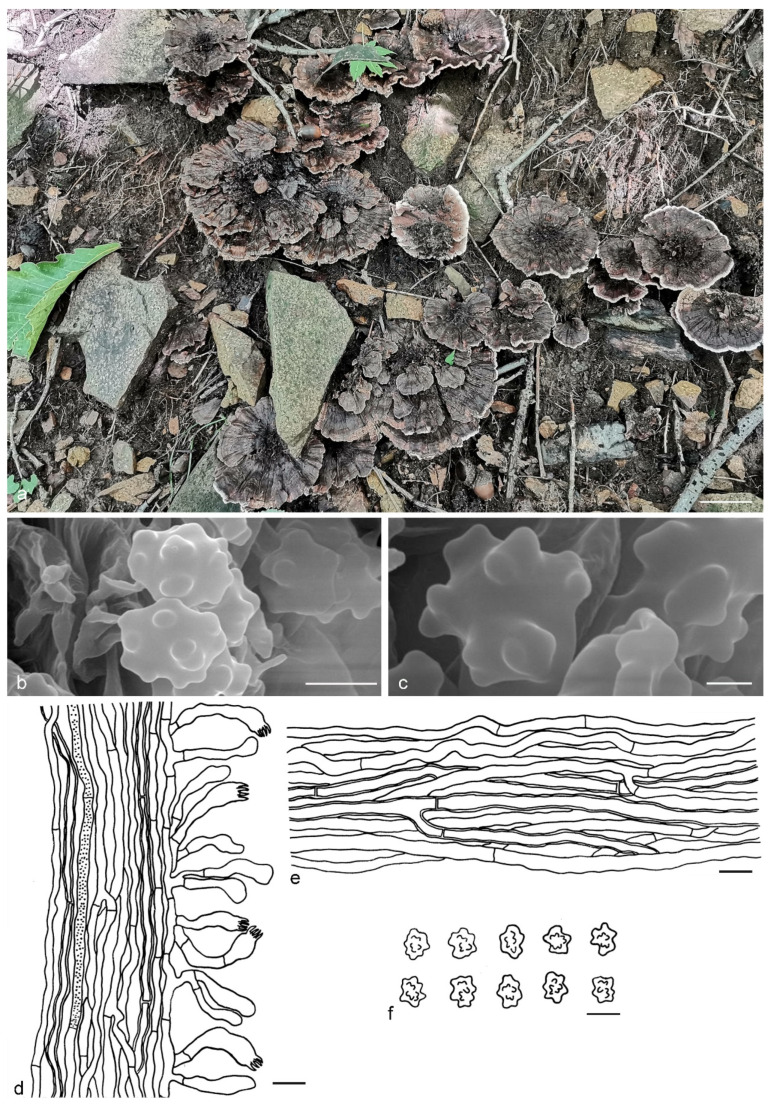
*Hydnellum sulcatum.* (**a**): Basidiocarps; (**b**,**c**): SEM of basidiospores; (**d**–**f**): Microscopic structures (drawn from IFP 019424); (**d**): Section of hymenophore trama with basidia; (**e**): Hyphae from pileus context; (**f**): Basidiospores. —Scale bars: (**a**) = 2 cm; (**b**) = 3 μm; (**c**) = 1 μm; (**d**,**e**) = 10 µm; (**f**) = 5 µm.

***Hydnellum sulcatum*** Y.H. Mu & H.S. Yuan, **sp. nov.** (Figure 12) 

MycoBank MB839043;

*Etymology*. *Sulcatum* (Latin), refers to the often grooved pileal surface.

Type: CHINA, Liaoning Province, Benxi County, Guanmenshan National Forest Park, on the ground of *Quercus* forest, 29 August 2020, *H. S. Yuan*, Yuan 14649 (holotype IFP 019424).

*Basidiocarps* annual, solitary to gregarious or multiple pilei overlapping and fused to form a compound cluster, soft and leathery when fresh, becoming woody and light in weight upon drying; taste mild, odor slightly fragrant when dry. *Pileus* subinfundibuliform when young, later applanate to flabelliform or circular with age, up to 65 mm diam and 3–6 mm thick at center. *Pileal surface* dark brown (9F4), obscurely concentrically zonate, often grooved, scabrous to fibrous when fresh, becoming fibrillose, rugose when dry; margin white (6A1) when fresh, light brown (6D5) when dry, even, sometimes lobed. *Spine surface* brown (7E7) when fresh, brown (6E5) to dark brown (7F5) when dry; spines up to 1.5 mm long, base up to 0.1 mm diam, conical, 5–7 per mm, more or less decurrent on stipe, without spines at pileus margin, brittle when dry. *Context* not duplex, up to 6 mm thick, brown (7E6), woody. *Stipe* lateral, up to 2 cm long and 1.5 cm diam, sometimes connate, leathery when fresh, woody upon drying, brown (7E5), pubescent, inside solid, cylindrical to flat or attenuate downwards with bulbous base when old. *Hyphal structure:* hyphal system monomitic; generative hyphae with simple-septa, CB+ in slightly thick-walled hyphae, IKI–; tissues olivaceous in KOH. *Context:* generative hyphae hyaline, thin-walled, moderately branched, simple-septate, straight, regularly arranged, sometimes flexuous and collapsed, mostly 4–6 μm diam. *Spines:* generative hyphae hyaline, thin to slightly thick-walled, moderately branched, more or less parallel along spines, often simple-septate, straight, 2–3 μm diam. *Cystidia* and cystidioles absent. *Basidia* clavate, thin-walled, with four sterigmata (2–3 μm long), simple-septate at base, 20–30 × 4–8 μm; basidioles similar to basidia. *Basidiospores* irregular ellipsoid to subglobose, brown, thin-walled, tuberculate, CB–, IKI–, (4–)4.1–5.8(–5.9) × (3.9–)4–4.6(–4.8) μm, Lm = 4.8 μm, Wm = 4.3 μm, Q = 1.14–1.19 (n = 60/2); tuberculi usually isolated, sometimes in groups of two or more, then bi- to trifurcate in shape, up to 0.9 μm long.

Additional specimens (paratypes) examined: CHINA, Liaoning Province, Kuandian County, Baishilazi National Nature Reserve, on the ground of *Quercus* forest, 8 August 2020, *H. S. Yuan*, Yuan 14521 (IFP 019425, paratype); Benxi County, Guanmenshan National Forest Park, on the ground of *Quercus* forest, 29 August 2020, *H. S. Yuan*, Yuan 14638 (IFP 019426, paratype); Yuan 14658 (IFP 019427, paratype); Yuan 14660 (IFP 019428, paratype).

Notes: *Hydnellum parvum* has a close phylogenetic relationship with *H. sulcatum*. The former species resembles the latter by compound fused pilei and rugulose pileal surface. However, the latter has a thinner stipe (0.7 × 0.2 cm vs. 2 × 1.5 cm in *H. sulcatum*), slightly longer spines (up to 2 mm vs. 1.5 mm in *H. sulcatum*) and shorter basidiospores (3–4 μm vs. 4.1–5.8 μm in *H. sulcatum*) [27]. *H. subsuccosum* resembles *H. sulcatum* by concentrically zonate pileus with similar size, context hyphae of similar width, the absence of clamp-connections and brown and subglobose basidiospores. However, *H. subsuccosum* differs by orange white to camel and nodulose or pitted pileal surface, longer spines (up to 3 mm vs. 1.5 mm in *H. sulcatum*), blue green to dark brown or black context tissue in KOH and longer basidia sterigmata (3–4 μm vs. 2–3 μm in *H. sulcatum*) [27]. *H. atrorubrum* is similar to *H. sulcatum* in having white to brown and even pileal margin and zonate pileal surface, olivaceous context tissues in KOH and basidiospores of similar shape with isolated or grouped tuberculi. However, *H. atrorubrum* can be differentiated by a flocculose to fibrillose or glabrescent pileal surface, longer spines (up to 3.5 mm vs. 1.5 mm in *H. sulcatum*) and basidia sterigmata (4–6 μm vs. 2–3 μm in *H. sulcatum*) and slightly longer tuberculi (up to 1.1 μm vs. 0.9 μm in *H. sulcatum*) of basidiospores. 

**Figure 13:**
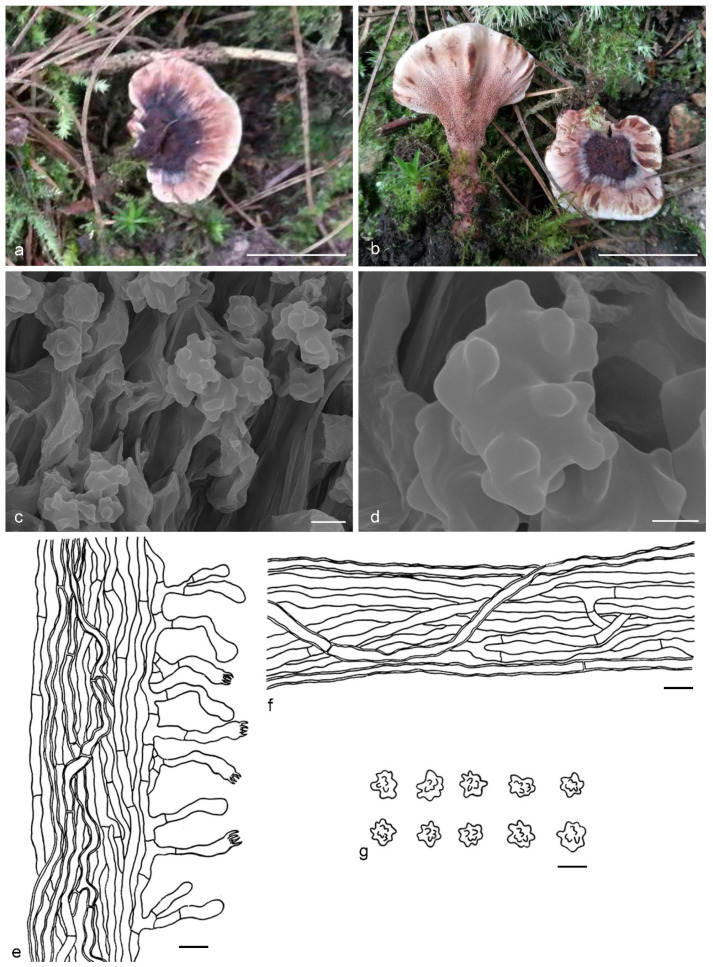
*Hydnellum yunnanense.* (**a**,**b**): Basidiocarps; (**c**,**d**): SEM of basidiospores; (**e**–**g**): Microscopic structures (drawn from IFP 019429); (**e**): Section of hymenophore trama with basidia; (**f**): Hyphae from pileus context; (**g**): Basidiospores. —Scale bars: (**a**,**b**) = 1 cm; (**c**) = 3 μm; (**d**) = 1 μm; (**e**,**f**) = 10 µm; g = 5 µm.

***Hydnellum yunnanense*** Y.H. Mu, X.H. Wang & H.S. Yuan, **sp. nov.** (Figure 13)

MycoBank MB839044

*Etymology*. *Yunnanense*, refers to the Yunnan Province, where the specimens were collected.

Type: CHINA, Yunnan Province, Nanjian County, Lingbaoshan National Forest Park, on the ground, 19 September 2019, *H. S. Yuan*, Yuan 14386 (holotype IFP 019429).

*Basidiocarps* annual, solitary to gregarious, soft and leathery when fresh, becoming woody and light in weight upon drying; taste mild, odor slightly fragrant when dry. *Pileus* subinfundibuliform when young, later flabelliform with age, up to 21 mm diam and 3–5 mm thick at center. *Pileal surface* grayish red (10D6) to dark brown (9F8), obscurely concentrically zonate, velutinate to tomentose when fresh, becoming rugulose to glabrescent when dry; margin white (6A1) when fresh, light brown (6D4) when dry, even, sometimes eroded with age. *Spine surface* white (10A1) to grayish red (106) when fresh, pale orange (5A3) to brown (6E7) when dry; spines up to 1.5 mm long, base up to 0.1 mm diam, conical, 5–8 per mm, more or less decurrent on stipe, without spines at pileus margin, brittle when dry. *Context* not duplex, up to 5 mm thick, reddish brown (8E4), woody. *Stipe* central to lateral, up to 4 cm long and 0.7 cm diam, sometimes connate, leathery when fresh, woody upon drying, brown (7E6), tomentose, solid inner, context with a dark line present at centre, cylindrical to attenuate downwards or broadening below with bulbous base when old. *Hyphal structure:* hyphal system monomitic; generative hyphae with simple-septa, CB+ in slightly thick-walled hyphae, IKI–; tissues olivaceous in KOH. *Context:* generative hyphae hyaline, thin- to slightly thick-walled, moderately branched, simple-septate, straight, regularly arranged, sometimes flexuous and collapsed, mostly 3–5 μm diam. *Spines:* generative hyphae hyaline, thin- to slightly thick-walled, frequently branched, more or less parallel along spines, often simple-septate, straight, 2–5 μm diam. *Cystidia* and cystidioles absent. *Basidia* clavate, thin-walled, with four sterigmata (2–4 μm long), simple-septate at base, 13–28 × 4–7 μm; basidioles similar to basidia. *Basidiospores* irregularly ellipsoid to subglobose, brown, thin-walled, tuberculate, CB–, IKI–, (4.1–)4.2–5.1(–5.3) × (3.4–)3.5–4.5(–5) μm, Lm = 4.7 μm, Wm = 4 μm, Q = 1.17–1.18 (n = 60/2); tuberculi usually isolated, sometimes in groups of two or more, then bi- to trifurcate in shape, up to 1.2 μm long.

Additional specimens (paratypes) examined: CHINA, Yunnan Province, Nanjian County, Lingbaoshan National Forest Park, on the ground, 19 September 2019, *H. S. Yuan*, Yuan 14396 (IFP 019430, paratype); Maguan County, Dalishu Township, Adushangba Village, on the ground, 7 August 2017, *S. F. Shi*, Shi 212 (IFP 019431, paratype).

Notes: The phylogenetic analyses support that *Hydnellum yunnanense* is sister to *H. sulcatum* (Figure 1). Morphologically, *H. sulcatum* resembles *H. yunnanense* in having an annual, solitary to gregarious basidiocarp with subinfundibuliform to flabelliform pileus with equal-length spines, a brown, pubescent, cylindrical stipe, olivaceous context tissue in KOH and basidiospores of similar shape. However, it differs from *H. yunnanense* in having a broader pileus (up to 65 mm vs. 21 mm in *H. yunnanense*) with scabrous to squamulose pileal surface and light brown pileal margin and shorter tuberculi (up to 0.9 μm vs. 1.2 μm in *H. yunnanense*). *H. rubidofuscum* resembles *H. yunnanense* by infundibuliform to flabelliform and concentrically zonate pileus, not duplex and woody context, thin- to slightly thick-walled and simple-septate hyphae in the context, basidia of similar width and basidiospores of similar shape and size. However, *H. rubidofuscum* differs by larger pileus (up to 70 mm vs. up to 21 mm in *H. yunnanense*) with reddish brown and scrupose, fibrillose to virgate pileal surface, grayish brown to reddish brown and longer spines (up to 3 mm vs. up to 1.5 mm in *H. yunnanense*) and thin-walled generative hyphae in the spines.

***Hydnellum*** **sp 3**

*Basidiocarps* annual, solitary. *Pileus* applanate, flabelliform. *Pileal surface* brown (7E7) to dark brown (7F7), concentrically zonate and pubescent. *Spines* reddish brown (9E6) and more or less decurrent, up to 1 mm long. *Stipe* lateral, cylindrical and slender. *Hyphal system* monomitic, generative hyphae simple-septa. *Basidia* clavate, with four sterigmata. *Basidiospores* irregularly subglobose, tuberculate, (4.8–)4.9–5.2(–5.3) × (4–)4.1–4.8(–5) μm.

Specimen examined: CHINA, Yunnan Province, Nanjian County, Lingbaoshan National Forest Park, on the ground of angiosperm forest, 19 September 2019, *H. S. Yuan*, Yuan 14388 (IFP 019438).

***Hydnellum* sp 4**

*Basidiocarps* annual, solitary to gregarious. *Pileus* irregularly flabelliform. *Pileal surface* grayish yellow (4B3) to yellowish brown (5E8), obscurely concentrically zonate and glabrescent. *Spines* yellowish brown (5D5) and more or less decurrent, up to 2 mm long. *Stipe* lateral and cylindrical. *Hyphal system* monomitic, generative hyphae simple-septa. *Basidia* clavate, with four sterigmata. *Basidiospores* irregularly subglobose, tuberculate, (4.1–)4.2–5.3(–6) × (4–)4.1–4.3(–4.8) μm.

Specimen examined: CHINA, Yunnan Province, Maguan County, Dalishu Township, on the ground in Fagaceous forest, 14 October 2017, *J. Wang*, Wang 295 (IFP 019439).

***Hydnellum*** **sp 5**

*Basidiocarps* annual, solitary. *Pileus* irregularly circular. *Pileal surface* zonate, velutinous and strigose with lobed margin. *Spines* decurrent, up to 1 mm long. *Stipe* lateral, cylindrical and broadened below with bulbous base when old. *Hyphal system* monomitic, generative hyphae simple-septa. *Basidia* clavate, with four sterigmata. *Basidiospores* irregularly ellipsoid to subglobose, tuberculate, (4–)4.1–5.5(–5.6) × (3–)3.1–4.7(–5.2) μm.

Specimen examined: CHINA, Liaoning Province, Xinbin County, Gangshan Nature Reserve, in the angiosperm forest dominated by *Quercus liaotungensis*, 26 August 2020, *H. S. Yuan*, Yuan 14594 (IFP 019440).

**Other** ***Hydnellum*** **species**

*Hydnellum complicatum*, *H. cumulatum*, *H. diabolus*, *H. geogenium*, *H. lundellii*, *H. martioflavus*, ***H. peckii***, *H. regium*, *H. versipelle*

***Hydnellum peckii*** Banker, in Peck, Bull. N.Y. St. Mus. 157: 28 (1912) (1911)

*Basidiocarps* single to concrescent. *Pileus* turbinate or elliptical, planar to subdepressed. *Pileal* *surface* white (6A1) to light orange (6A4), colliculose, rarely scrobiculate and glabrous. *Spines* brownish orange (7C4), decurrent, up to 3 mm long. *Stipe* central and terete. *Hyphal system* monomitic; generative hyphae mostly with clamp-connections, minority of simple-septa. *Basidia* clavate, with simple-septate at base and four sterigmata. *Basidiospores* irregularly subglobose, tuberculate, (4.1–)4.2–5.1(–5.3) × (3.8–)3.9–4.4(–4.6) μm.

*Specimens examined.* CHINA, Xizang Autonomous Region, Bomi County, on the ground in *Pinus* mixed forest, 19 July 2019, *H. S. Yuan*, Yuan 13708 (IFP 0193433); Yuan 13720 (IFP 019434).

Notes: The studied samples were found in high altitude *Pinus* mixed forest. These two specimens clustered with two other samples (SSvantesson328 and ELarsson174-14) of *Hydnellum peckii* from Europe with full support (100% in ML and 1.00 BPP) (Figure 1). The ITS+LSU sequence identity between the specimens from China and Europe was 0.99 in Bioedit pairwise alignment. In morphology, the characters of our specimens overlap with those of *H. peckii* [1,27]. Therefore, we introduce *H. peckii* as a new record to China.
**Key to species of *Hydnellum* from China**1. Basidiocarps fleshy21.→ Basidiocarps woody42. Pileal surface not scaled*H. coactum*2.→ Pileal surface scaled33. Pileal surface with ascend squama*H. grosselepidotum*3.→ Pileal surface with appressed squama*H. lidongensis*4. Context tissue olivaceous in KOH54.→ Context tissue blue-green in KOH*H. peckii*5. Hyphae with clamp-connections in the context65.→ Hyphae without clamp-connections in the context and spines86. Basidiocarps with dark violet spines underneath pileus*H. atrospinosum*6.→ Basidiocarps with different colored spines underneath pileus77. Pileal surface fibrillose, rugose when dry, spines up to 1.5 mm long*H. fibulatum*7.→ Pileal surface pitted, colliculose when dry, spines up to 6 mm long*H. caeruleum*8. Inflated hyphae present from the context98.→ Inflated hyphae absent in the context109. Generative hyphae mostly inflated in the context, pileal surface scrobiculate when dry*H. inflatum*9.→ Generative hyphae occasionally inflated in the context, pileal surface granulose when dry*H. granulosum*10. Stipe thin, rhizomorphs-like*Hydnellum* sp 110.→ Stipe cylindrical to flattened1111. Pileal surface colored brownish orange to brownish red*H. brunneorubrum*11.→ Pileal surface differently colored1212. Pileal margin involute and wavy, sometimes lobed or rimose1312.→ Pileal margin even or effused, sometimes lobed or eroded1413. Pileal surface glabrescent*Hydnellum* sp 413.→ Pileal surface not glabrescent1514. Pileal surface azonate and spongy1614.→ Pileal surface obsurely concentrically zonate to zonate and not spongy1715. Pileal surface floccose to squamulose when fresh, context corky*H. squamulosum*15.→ Pileal surface tomentose and scrupose when fresh, context woody*H. bomiense*16. Basidiocarps single to gregarious and stipe single and long*H. spongiosipes*16.→ Basidiocarps coalescent and stipe connate and short*Hydnellum* sp 217. Stipe context with a dark line at centre1817.→ Stipe context without a dark line at centre1918. Pileus and spines grayish red*H. yunnanense*18.→ Pileus brown and spines reddish brown*Hydnellum* sp 319. Spines up to 3 or 3.5 mm long2019.→ Spines up to 1 or 1.5 mm long2120. Pileus light brown to dark ruby*H. atrorubrum*20.→ Pileus reddish brown*H. rubidofuscum*21. Basidiocarps gregarious or multiple pilei overlapping and pileal surface often grooved, scabrous to fibrous*H. sulcatum*21.→ Basidiocarps solitary and pileal surface velutinous and strigose*Hydnellum* sp 5

## 5. Discussion

Baird et al. [27] constructed a phylogenetic tree of Bankeraceae using ITS sequences of specimens from the temperate southeastern United States, and suggested that neither *Hydnellum* nor *Sarcodon* were monophyletic. In the current study, phylogenetic analyses using four loci (nLSU + ITS + SSU + RPB2) of *Hydnellum* and *Sarcodon* were carried out using sequences from Europe, Asia and America. Our results also verify that *Sarcodon* is paraphyletic; with regard to *Hydnellum*, some species of *Sarcodon* nesting among *Hydnellum* species. Larsson et al. [31] provided phylogenetic analyses on *Hydnellum* and *Sarcodon* based on ITS and LSU sequences, confirming that *Sarcodon* makes *Hydnellum* paraphyletic and the genus limits were revised, 12 species of *Sarcodon* moved to *Hydnellum.* Morphologically, they proposed to use the size of the basidiospores to separate the two genera, delimiting *Hydnellum* and *Sarcodon* species with basidiospore lengths in the ranges of 4.45–6.95 µm and 7.4–9 µm, respectively. These results indicate that the traditional classification system based on the consistency of *Sarcodon* (fleshy) and *Hydnellum* (woody) did not conform to the monophyletic concept of the two genera.

Based on microscopical hyphal structure species of *Hydnellum* and *Sarcodon* can be divided into five groups, as shown in Table 3: (I) Context of the pileus and the spines composed of simple-septate hyphae (includes *H. aurantiacum*, *H. auratile* and 49 other taxa); (II) Pileus composed mostly of simple-septate hyphae with occasional clamp-connections and spinal trama composed of simple-septate hyphae (includes *H. caeruleum*, *H. ferrugipes* and *H. fibulatum*); (III) Both pileus and the spines composed of predominantly clamped hyphae with occasional simple-septa (includes *H. peckii* and *H. versipelle*); (IV) Pileus composed of only hyphae with clamp-connections and spine with only simple-septate hyphae (includes *H. diabolus*); (V) Both pileus and the spines composed of clamped hyphae (includes *H. atrospinosum*, *H. cyanopodium*, *H. geogenium*, *H. scleropodium*, *H. suaveolens*, *S. aspratus*, *S. imbricatus*, *S. leucopus*, *S. quercinofibulatus*, *S. scabripes* and *S. squamosus*). One species, *H. regium*, has not been classified into these five groups because the information is not available from the original description [33].

In the phylogenetic tree, ten subgenera with moderate to high support in *Hydnellum* have been distinguished (Figure 1), and each subgenus possesses distinctive morphological characteristics. Group I can be categorized into seven subgenera. The subgenus *Croceum* has an orange basidiocarps and includes *H. aurantiacum*, *H. auratile*, *H. brunneorubrum*, *H. chrysinum* and *H. earlianum*; the subgenus *Inflatum* has the appearance of inflated hypha in the context of the pileus and includes *H. cristatum*, *H. granulosum*, *H. inflatum*, *H. mirabile* and *H. piperatum*; the subgenus *Rhizomorphum* has rhizomorphs-like stipe and includes *H. gracilipes* and *Hydnellum* sp 1; the subgenus *Scabrosum* has pileal surface with scabrosity and includes *H. amygdaliolens*, *H. coactum*, *H. fagiscabrosum*, *H. fennicum*, *H. grosselepidotum*, *H. illudens*, *H. lepidum*, * H. lidongensis*, *H. nemorosum*, *H. scabrosellum*, *H. scabrosum* and *H. underwoodii*; the subgenus *Spongiosum* corresponds to a spongy pileal surface and includes *H. ferrugineum*, *H. pineticola*, *H. spongiosipes* and *Hydnellum* sp 2; the subgenus *Violaceum* corresponds to the violaceous basidiocarps and includes *H. fuligineoviolaceum*, *H. fuscoindicum*, *H. glaucopus*, *H. joeides* and *H. roseoviolaceum*; the subgenus *Zonatum* has a concentrically zonate pileal surface and includes *H. atrorubrum*, *H. bomiense*, *H. concrescens*, *H. dianthifolium*, *H. parvum*, *H. rubidofuscum*, *H. scrobiculatum*, *H. squamulosum*, *H. subsuccosum*, *H. sulcatum*, *H. yunnanense*, *Hydnellum* sp 3, *Hydnellum* sp 4 and *Hydnellum* sp 5. Group II corresponds to the subgenus *Subindufibulatum*, having occasionally clamped hyphae in the context of the pileus, and includes *H. caeruleum*, *H. ferrugipes* and *H. fibulatum*. Group V corresponds to all hyphae clamped in the pileus and the spines, incorporating two subgenera of *Hydnellum* and six *Sarcodon* species; the subgenus *Hydnellum* has dark spines and includes *H. atrospinosum* and *H. suaveolens*; the subgenus *Caesispinosum* has blue spines and includes *H. cyanopodium* and *H. scleropodium*; *H. geogenium* is morphologically related to other species in Group V, but is distantly related in the phylogenetic tree; *Sarcodon* clade *S. aspratus*, *S. imbricatus*, *S. leucopus*, *S. quercinofibulatus*, *S. scabripes* and *S. squamosus* have the characteristics of long spores compared with the *Hydnellum* species. Therefore, the classification system using hyphal structure type and phylogenetic subgenera can fix the positions for most species in *Hydnellum* and *Sarcodon*, except for some species in Groups III, IV and those without phylogenetic support.

The specimens involved in this study were collected from the northeast, northwest and southwest regions of China, where industrial pollution is relatively low and vegetation is relatively abundant. The forests are primarily dominated by Pinaceae and Fagaceae trees such as *Pinus* spp., *Picea* spp., *Quercus* spp., *Lithocarpus* spp. and a small portion of other tree families. Thus, we speculated that these species may form an ectomycorrhizal association with Pinaceae and Fagaceae host trees. The species diversity and basidiocarps richness of stipitate hydnoid fungi represented by *Hydnellum* and *Sarcodon* species have shown a declining trend across Europe and some regions of North America during the 1970s to 2000s [6,55,56,57]; the phenomenon is most probably caused by forest management, air pollutants, forest soil acidification, nitrogen deposition and forest succession, among other causes [19,58,59,60,61]. Many stipitate hydnoid fungi have been included in national Red Lists in Europe [27,62,63,64]. With the rapid industrialization in China over the past four decades, a significant decline of basidiocarps has also been observed during the course of our field investigation. The identification and description of stipitate hydnoid fungi in this paper will contribute to the understanding of species diversity and provide baseline data for the evaluation and protection of these fungi in China.

## Figures and Tables

**Table 1:** Voucher numbers, geographic origins and GenBank accession numbers for the specimens included; sequences produced in this study are in bold.

Species	Geographic Origin	Voucher Number	GenBank Accessions No.			
			SSU	ITS	nLSU	RPB2
*Amaurodon aquicoeruleu* Agerer	Australia	UK452	–	AM490944	AM490944	–
*A. sumatranus* Miettinen & Kõljalg	Indonesia	O. Miettinen5877	–	AM490943	–	–
*A. viridis* (Alb. & Schwein.) J. Schröt.	Norway	KHLarsson14947b	–	MK602707	MK602707	–
*A. viridis*	Russia	TAA149664	–	AM490942	AY586625	–
*Bankera fuligineoalba* (J.C. Schmidt) Coker & Beers ex Pouzar	Sweden	ELarsson400-13	–	MK602708	MK602708	–
*B. fuligineoalba*	Estonia	TAA152454	–	–	AY586635	–
*B. violascens* (Alb. & Schwein.) Pouzar	Finland	MVijanen130902	–	MK602709	MK602709	–
*B. violascens*	–	RGC14-033	–	MH310793	–	–
*Boletopsis grisea* (Peck) Bondartsev & Singer	Sweden	UPS F-120382	–	MN536751	MN535646	–
*B. grisea*	Spain	AH 42971	–	MN536747	MN535642	–
*B. leucomelaena* (Pers.) Fayod	Sweden	Krikorev140912	–	MK602710	MK602710	–
*B. nothofagi* J.A. Cooper & P. Leonard	New Zealand	PDD:96007	–	JQ417193	–	–
*Hydnellum amygdaliolens* (Rubio Casas, Rubio Roldán & Català) E. Larss., K.H. Larss. & Kõljalg	Iberian Peninsula	SC-2011	–	JN376763	–	–
***H. atrorubrum***	**China**	**Wei8315**	**–**	**MW579937**	**–**	**–**
***H. atrorubrum***	**China**	**Wei8261**	**MW579910**	**MW579936**	**MW579884**	**–**
***H. atrospinosum***	**China**	**Yuan6495**	**MW579911**	**MW579938**	**MW579885**	**–**
***H. atrospinosum***	**China**	**Yuan6514**	**MW579913**	**MW579940**	**MW579886**	**–**
***H. atrospinosum***	**China**	**Yuan6520**	**MW579912**	**MW579939**	**–**	**–**
*H. aurantiacum* (Batsch) P. Karst.	Norway	EBendiksen177-07	–	MK602712	MK602712	–
*H. aurantiacum*	Norway	OF29502	–	MK602713	MK602713	–
*H. auratile* (Britzelm.) Maas Geest.	Norway	OF242763	–	MK602715	MK602715	–
*H. auratile*	Norway	OF294095	–	MK602714	MK602714	–
***H. bomiense***	**China**	**Yuan 13759**	**MW579914**	**MW579941**	**MW579887**	**OK254206**
***H. bomiense***	**China**	**Yuan 13767**	**MW579915**	**MW579942**	**–**	**–**
*H. bomiense*	Estonia	TUF100611	–	UDB003287	–	–
*H. bomiense*	Costa Rica	TUF100057	–	UDB003286	–	–
***H. brunneorubrum***	**China**	**Yuan12997**	**MW579917**	**MW579944**	**MW579889**	**OK254217**
***H. brunneorubrum***	**China**	**Yuan14339**	**MW579916**	**MW579943**	**MW579888**	**OK254216**
***H. brunneorubrum***	**China**	**Yuan14668**	**MW579918**	**MW579945**	**MW579890**	**OK254218**
*H. caeruleum* (Hornem.) P. Karst.	Norway	EBendiksen584-11	–	MK602719	MK602719	–
*H. caeruleum*	Norway	EBendiksen575-11	–	MK602718	MK602718	–
***H. caeruleum***	**China**	**Wei1474a**	**–**	**MW579965**	**–**	**–**
*H. chrysinum* K.A. Harrison	–	SC071	–	KJ534291	–	–
*H. coactum* Y.H. Mu & H.S. Yuan	China	Wei8094	–	MN846278	MN846287	–
*H. coactum*	China	Shi181	–	MN846279	MN846288	–
*H. complicatum* Banker	USA	REB-71	–	KC571711	–	–
*H. complicatum*	USA	REB-329	–	KC571712	–	–
*H. concrescens* (Pers.) Banker	USA	SEW 88	–	AY569025	–	–
*H. concrescens*	Mexico	GO-2009-204	–	KC152116	–	–
*H. cristatum* (Bres.) Stalpers	USA	REB-169	–	JN135174	–	–
*H. cristatum*	USA	REB-88	–	KC571718	–	–
*H. cumulatum* K.A. Harrison	Finland	TU115384	–	UDB011871	UDB011871	–
*H. cumulatum*	Estonia	TU111191	–	UDB032402	–	–
*H. cyanopodium* K.A. Harrison	USA	SEW 85	–	AY569027	–	–
*H. diabolus* Banker	Canada	KAH13873	–	AF351863	–	–
*H. dianthifolium* Loizides	Cyprus	ML61211HY	–	KX619419	–	–
*H. dianthifolium*	Italy	ML902162HY	–	KX619420	–	–
*H. earlianum* Banker	USA	REB-75	–	KC571724	–	–
*H. earlianum*	USA	REB-375	–	JN135179	–	–
*H. fagiscabrosum* A.M. Ainsw. & Nitare	Sweden	GB-0195621	–	MW144293	MW144293	–
*H. fagiscabrosum*	Sweden	GB-0195622	–	MW144296	MW144296	–
*H. fennicum* (P. Karst.) E. Larss, K.H. Larss. & Kõljalg	Norway	OF242833	–	MK602738	MK602738	–
*H. fennicum*	Norway	OF294087	–	MK602737	MK602737	–
*H. ferrugineum* (Fr.) P. Karst.	Norway	OF297319	–	MK602720	MK602720	–
*H. ferrugineum*	Sweden	ELarsson197-14	–	MK602722	MK602722	–
*H. ferrugipes* Coker	USA	REB-176	–	KC571727	–	–
*H. ferrugipes*	USA	REB-68	–	JN135176	–	–
***H. fibulatum***	**China**	**Yuan14646**	**MW579926**	**MW579957**	**–**	**–**
***H. fibulatum***	**China**	**Yuan14656**	**MW579927**	**MW579958**	**–**	**–**
*H. fuligineoviolaceum* (Kalchbr.) E. Larss., K.H. Larss. & Kõljalg	Sweden	BNylen130918	–	MK602741	MK602741	–
*H. fuligineoviolaceum*	Sweden	LA120818	–	MK602740	MK602740	–
*H. fuscoindicum* (K.A. Harrison) E. Larss., K.H. Larss. & Kõljalg	USA	OSC 113641	–	EU669230	EU669280	–
*H. fuscoindicum*	USA	OSC 107844	–	EU669229	EU669279	–
*H. glaucopus* (Maas Geest. & Nannf.) E. Larss., K.H. Larss. & Kõljalg	Sweden	JNitare060916	–	MK602744	MK602744	–
*H. glaucopus*	Sweden	Edvinson110926	–	MK602745	MK602745	–
*H. geogenium* (Fr.) Banker	–	AFTOL-ID 680	AY752971	DQ218304	AY631900	DQ408133
*H. geogenium*	Norway	OF296213	–	MK602724	MK602724	–
*H. gracilipes* (P. Karst.) P. Karst.	Sweden	ELarsson219-11	–	MK602726	MK602726	–
*H. gracilipes*	Sweden	GB-0113779	–	MK602727	MK602727	–
***H. granulosum***	**China**	**Yuan12213a**	**MW579921**	**MW579948**	**MW579893**	**OK254213**
***H. granulosum***	**China**	**Yuan12213b**	**MW579920**	**MW579947**	**MW579892**	**OK254212**
*H. grosselepidotum* Y.H. Mu & H.S. Yuan	China	Wei8120	–	MN846274	MN846283	–
*H. grosselepidotum*	China	Wei8075	–	MN846276	MN846285	–
*H. illudens* (Maas Geest.) Nitare	Sweden	GB-0195819	–	MW144341	MW144341	–
*H. illudens*	Norway	O-F-242769	–	MW144335	MW144335	–
***H. inflatum***	**China**	**Wang80**	**MW579922**	**MW579949**	**MW579894**	**OK254210**
***H. inflatum***	**China**	**Shi506**	**MW579923**	**MW579950**	**MW579895**	**OK254211**
*H. joeides* (Pass.) E. Larss., K.H. Larss. & Kõljalg	Sweden	KHjortstam17589	–	MK602750	MK602750	–
*H. joeides*	Sweden	Nitare110829	–	MK602751	MK602751	–
*H. lepidum* (Maas Geest.) E. Larss., K.H. Larss. & Kõljalg	Sweden	JNitare110829	–	MK602754	MK602754	–
*H. lepidum*	Sweden	RGCarlsson10-065	–	MK602752	MK602752	–
*H. lidongensis* Y.H. Mu & H.S. Yuan	China	We8365	–	MN846280	MN846289	–
*H. lidongensis*	China	Wei8329	–	MN846281	MN846290	–
*H. lundellii* (Maas Geest. & Nannf.) E. Larss., K.H. Larss. & Kõljalg	Norway	OF242639	–	MK602759	MK602759	–
*H. lundellii*	Norway	OF295814	–	MK602760	MK602760	–
*H. martioflavum* (Snell, K.A. Harrison & H.A.C. Jacks.) E. Larss., K.H. Larss. & Kõljalg	Norway	OF242435	–	MK602762	MK602762	–
*H. martioflavum*	Norway	OF242872	–	MK602761	MK602761	–
*H. mirabile* (Fr.) P. Karst.	Sweden	SLund140912	–	MK602730	MK602730	–
*H. mirabile*	Sweden	ELarsson170-14	–	MK602729	MK602729	–
*H. nemorosum* A.M. Ainsw. & E. Larss.	Norway	O-F-242352	–	MW144372	MW144372	–
*H. nemorosum*	Sweden	GB-0195631	–	MW144373	MW144373	–
*H. parvum* Banker	USA	REB-131	–	JN135187	–	–
*H. parvum*	USA	REB-392	–	KC571717	–	–
*H. peckii* Banker	Norway	SSvantesson328	–	MK602731	MK602731	–
*H. peckii*	Sweden	ELarsson174-14	–	MK602732	MK602732	–
***H. peckii***	**China**	**Yuan13708**	**MW579931**	**MW579966**	**MW579905**	**OK254214**
***H. peckii***	**China**	**Yuan13720**	**MW579932**	**MW579967**	**MW579906**	**OK254215**
*H. pineticola* K.A. Harrison	USA	REB-49	–	KC571733	–	–
*H. pineticola*	USA	REB-43	–	JN135175	–	–
*H. piperatum* Coker ex Maas Geest.	USA	REB-332	–	JN135173	–	–
*H. piperatum*	USA	REB-304	–	KC571723	–	–
*H. regium* K.A. Harrison	USA	SEW 93	–	AY569031	–	–
*H. roseoviolaceum* Nitare	Sweden	GB-0195936	–	MW144374	MW144374	–
*H. roseoviolac* *eum*	Sweden	GB-0195687	–	MW144375	MW144375	–
***H. rubidofuscum***	**China**	**Yuan14561**	**MW579924**	**MW579951**	**MW579896**	**OK254207**
***H. rubidofuscum***	**China**	**Yuan14587**	**MW579925**	**MW579952**	**MW579897**	**OK254208**
***H. rubidofuscum***	**China**	**Yuan14654**	**–**	**MW579953**	**MW579898**	**OK254209**
*H. scabrosum* (Fr.) E. Larss., K.H. Larss. & Kõljalg	Norway	OF360777	–	MK602765	MK602765	
*H. scabrosum*	Norway	OF292320	–	MK602766	MK602766	
*H. scabrosellum* Nitare	Sweden	GB-0195792	–	MW144380	MW144380	–
*H. scabrosellum*	Sweden	GB-0195807	–	MW144381	MW144381	–
*H. scleropodium* K.A. Harrison	USA	REB-3	–	JN135186	–	–
*H. scleropodium*	USA	REB-352	–	KC571740	–	–
*H. scrobiculatum* (Fr.) P. Karst.	USA	REB-78	–	JN135181	–	–
*H. spongiosipes* (Peck) Pouzar	USA	REB-107	–	KC571743	–	–
*H. spongiosipes*	USA	REB-52	–	JN135184	–	–
***H. spongiosipes***	**China**	**Yuan14517**	**MW579933**	**MW579968**	**MW579907**	**OK254219**
***H. squamulosum***	**China**	**Yuan13615**	**–**	**MW579954**	**–**	**–**
***H. squamulosum***	**China**	**Yuan13625**	**–**	**MW579956**	**MW579899**	**OK254204**
***H. squamulosum***	**China**	**Yuan13743**	**–**	**MW579955**	**–**	**OK254203**
*H. suaveolens* (Scop.) P. Karst.	Sweden	ELarsson8-14	–	MK602735	MK602735	–
*H. suaveolens*	Norway	SSvantesson877	–	MK602736	MK602736	–
*H. subsuccosum* K.A. Harrison	USA	SEW 55	–	AY569033	–	–
*H. subsuccosum*	USA	REB-10	–	JN135178	–	–
***H. sulcatum***	**China**	**Yuan14521**	**MW579930**	**MW579961**	**MW5** **79902**	**OK254202**
***H. sulcatum***	**China**	**Yuan14649**	**MW579929**	**MW579960**	**MW579901**	**–**
***H. sulcatum***	**China**	**Yuan14660**	**MW579928**	**MW579959**	**MW579900**	**OK254201**
***H. yunnanens*** ***e***	**China**	**Yuan14386**	**–**	**MW579962**	**MW579903**	**OK254199**
***H. yunnanens*** ***e***	**China**	**Yuan14396**	**–**	**MW579963**	**MW579904**	**OK25420** **0**
***H. yunnanens*** ***e***	**China**	**Shi212**	**–**	**MW579964**	**–**	**–**
*H. underwoodii* (Banker) E. Larss., K.H. Larss. & Kõljalg	USA	REB-358	–	JN135189	–	–
*H. underwoodii*	USA	REB-119	–	KC571782	–	–
*H. versipelle* (Fr.) E. Larss., K.H. Larss. & Kõljalg	Sweden	RGCarlsson13-057	–	MK602771	MK602771	–
*H. versipelle*	Sweden	RGCarlsson11-08	–	MK602772	MK602772	–
***Hydnellum* sp 1**	**China**	**Shi164**	**–**	**MW579969**	**–**	**–**
***Hydnellum* sp 2**	**China**	**Yuan14387**	**MW579934**	**MW579970**	**MW579908**	**–**
***Hydnellum* sp 3**	**China**	**Yuan14388**	**–**	**MW579971**	**–**	**–**
***Hydnellum* sp 4**	**China**	**Wang2** **95**	**–**	**MW579972**	**–**	**–**
***Hydnellum* sp 5**	**China**	**Yuan14594**	**MW579935**	**MW579973**	**MW579909**	**OK254205**
*Lenzitopsis daii* L.W. Zhou & Kõljalg	China	Yuan 2959	–	JN169799	JN169795	–
*L. daii*	China	Yuan2952	–	JN169798	JN169794	–
*L. oxycedri* Malençon & Bertault	Spain	KHLarsson15304	–	MK602774	MK602774	–
*L. oxycedri*	–	UK 635	–	JN169800	JN169796	–
*Odontia fibrosa* (Berk. & M.A. Curtis) Kõljalg	China	TU115028	–	MK602775	MK602775	
*O. fibrosa*	China	LL_17	–	MT678878	–	–
*O. sparsa* Yuan, Y.C. Dai & H.S. Yuan	China	Yuan10718	–	MG719980	–	–
*O. sparsa*	China	Yuan10780	–	MG719979	–	–
*Phellodon* cf. *niger*	Sweden	ELarsson35-14	–	MK602782	MK602782	–
*P. tomentosus* (L.) Banker	Norway	EBendiksen11-810	–	MK602781	MK602781	–
*P. tomentosus*	–	BG Thesis	–	–	AF518637	–
*Polyozellus mariae* Voitk & Kõljalg	Canada	TU117348	–	MF100831	MF100831	–
*P. mariae*	Canada	TU117235	–	MF100826	–	–
*P. multiplex* (Underw.) Murrill	USA	TU117350	–	MF100830	MF100830	–
*P. multiplex*	China	TU115049	–	MF100812	MF100812	–
*Pseudotomentella abundiloba* Svantesson	Norway	OF110312		MK290731	MK290731	
*P. flavovirens* (Höhn. & Litsch.) Svrček	Finland	KHLarsson16190	–	MK602780	MK602780	–
*P. rotundispora* Svantesson	Sweden	SS394	–	MK290728	MK290728	–
*P. rotundispora*	Sweden	SS413	–	MK290674	–	–
*P. umbrinascens* Svantesson	Sweden	SS335	–	MK290697	MK290697	–
*Sarcodon aspratus* (Berk.) S. Ito	–	–	–	DQ448877	–	–
*S. aspratus*	–	–	–	AF335110	–	–
*S. imbricatus* (L.) P. Karst.	Norway	SSvantesson355	–	MK602748	MK602748	–
*S. imbricatus*	Sweden	ELarsson384-10	–	MK602747	MK602747	–
*S. leucopus* (Pers.) Maas Geest. & Nannf.	Norway	OF296099	–	MK602755	MK602755	–
*S. leucopus*	Sweden	PHedberg080811	–	MK602757	MK602757	–
*S.quercinofibulatus* Pérez-De-Greg., Macau & J. Carbó	Italy	JC-20090718.2	–	JX271818	MK602773	–
*S. quercinofibulatus*	USA	TENN	–	MG663244	–	–
*S. scabripes* (Peck) Banker	Mexico	FCME:23240	–	EU293829	–	–
*S. scabripes*	USA	REB-351	–	JN135191	–	–
*S.squamosus* (Schaeff.) P. Karst.	Norway	OF295554	–	MK602769	MK602769	–
*S. squamosus*	Norway	OF177452	–	MK602768	MK602768	–
*Steccherinum murashkinskyi* (Burt) Maas Geest.	Russia	X449	–	JN710588	JN710588	–
*S. ochraceum* (Pers. ex J.F. Gmel.) Gray	Sweden	KHL11902	–	JQ031130	JQ031130	–
*Thelephora ganbajun* M. Zang	China	GDGM 48899	–	MF593267	MH620355	–
*T. ganbajun*	China	GDGM 48891	–	MF593266	MH620354	–
*T. iqbalii* Nasir & Hanif	Pakistan	MH810	–	JX241471	–	–
*T. terrestris* Ehrh.	Denmark	DMS-9327942	–	MT644883	MT644883	–
*T. terrestris*	Norway	ELarsson295-13	–	MK602777	MK602777	–
*Tomentella fuscocrustosa* H.S. Yuan, X. Lu & Y.C. Dai	China	Yuan11399	–	MK211712	MK446366	–
*T. fuscocrustosa*	China	Yuan11420	–	MK211713	MK446367	–
*T.patagonica* Kuhar & Rajchenb.	Argentina	BAFC52372	–	KT032090	KT032102	–
*T. patagonica*	Argentina	BAFC52373	–	KT032091	KT032103	–
*Tomentellopsis bresadoliana* (Sacc. & Trotter) Jülich & Stalpers	Sweden	JEH 031011	–	EU118674	EU118674	–
*T. pulchella* Kõljalg & Bernicchia	Norway	KHLarsson16366	–	MK602779	MK602779	–

Newly generated sequences in this study are in bold.

**Table 2:** The gene fragments, their corresponding primers and primer sequences used in this study.

Genes	Primers	Primer Sequences (5’-3’)	References
nLSU	LROR	ACCCGCTGAACTTAAGC	Vilgalys & Hester 1990 [48]
	LR7	TACTACCACCAAGATCT	Vilgalys & Hester 1990 [48]
ITS	ITS1-F	CTTGGTCATTTAGAGGAAGTAA	White et al. 1990 [49]
	ITS4	TCCTCCGCTTATTGATATGC	White et al. 1990 [49]
nSSU	NS1	GTAGTCATATGCTTGTCTC	White et al. 1990 [49]
	NS4	CTTCCGTCAATTCCTTTAAG	White et al. 1990 [49]
RPB2	bRPB2-6F	TGGGGYATGGTNTGYCCYGC	Liu et al. 1999 [50]
	bRPB2-7.1R	CCCATRGCYTGYTTMCCCATDGC	Liu et al. 1999 [50]

**Table 3:** Hyphal-septa type observations in the context of the pileus and the spine trama in the species of *Hydnellum* and *Sarcodon*.

Group	Subgenus	Species	Pileus	Spines	References
**I**	***Croceum***	*Hydnellum aurantiacum*	simple-septa	simple-septa	Maas Geesteranus 1975 [1]
		*H. auratile*	simple-septa	simple-septa	Maas Geesteranus 1971 [26]
		***H. brunneorubrum***	simple-septa	simple-septa	In this study
		*H. chrysinum*	simple-septa	simple-septa	Baird 1986 [51]
		*H. earlianum*	simple-septa	simple-septa	Baird et al. 2013 [27]
	***Inflatum***	*H. cristatum*	simple-septa	simple-septa	Baird et al. 2013 [27]
		***H. granulosum***	simple-septa	simple-septa	In this study
		***H. inflatum***	simple-septa	simple-septa	In this study
		*H. piperatum*	simple-septa	simple-septa	Baird et al. 2013 [27]
		*H. mirabile*	simple-septa	simple-septa	Maas Geesteranus 1975 [1]
	***Rhizomorphum***	*H. gracilipes*	simple-septa	simple-septa	Koljalg & Renvall 2000 [35]
		***Hydnellum* sp 1**	simple-septa	simple-septa	In this study
	***Scabrosum***	*H. amygdaliolens*	simple-septa	simple-septa	Rubio Casas et al. 2011 [52]
		*H. coactum*	simple-septa	simple-septa	Mu et al. 2020 [38]
		*H. fagiscabrosum*	simple-septa	simple-septa	Nitare et al. 2021 [53]
		*H. fennicum*	simple-septa	simple-septa	Hahn et al. 2018 [54]
		*H. grosselepidotum*	simple-septa	simple-septa	Mu et al. 2020 [38]
		*H. illudens*	simple-septa	simple-septa	Nitare et al. 2021 [53]
		*H. lepidum*	simple-septa	simple-septa	Maas Geesteranus 1975 [1]
		*H. lidongensis*	simple-septa	simple-septa	Mu et al. 2020 [38]
		*H. nemorosum*	simple-septa	simple-septa	Nitare et al. 2021 [53]
		*H. scabrosellum*	simple-septa	simple-septa	Nitare et al. 2021 [53]
		*H. scabrosum*	simple-septa	simple-septa	Baird et al. 2013 [27]
		*H. underwoodii*	simple-septa	simple-septa	Baird et al. 2013 [27]
	***Spongiosum***	*H. ferrugineum*	simple-septa	simple-septa	Baird et al. 2013 [27]
		*H. pineticola*	simple-septa	simple-septa	Baird et al. 2013 [27]
		***H. spongiosipes***	simple-septa	simple-septa	Baird et al. 2013 [27]
		***Hydnellum* sp 2**	simple-septa	simple-septa	In this study
	***Violaceum***	*H. fuligineoviolaceum*	simple-septa	simple-septa	Maas Geesteranus 1971 [26]
		*H. fuscoindicum*	simple-septa	simple-septa	Maas Geesteranus 1967 [65]
		*H. glaucopus*	simple-septa	simple-septa	Maas Geesteranus 1975 [1]
		*H. joeides*	simple-septa	simple-septa	Baird et al. 2013 [27]
		*H. roseoviolaceum*	simple-septa	simple-septa	Nitare et al. 2021 [53]
	***Zonatum***	***H. atrorubrum***	simple-septa	simple-septa	In this study
		***H. bomiense***	simple-septa	simple-septa	In this study
		*H. concrescens*	simple-septa	simple-septa	Baird et al. 2013 [27]
		*H. dianthifolium*	simple-septa	simple-septa	Loizides et al. 2016 [36]
		*H. parvum*	simple-septa	simple-septa	Baird et al. 2013 [27]
		***H. rubidofuscum***	simple-septa	simple-septa	In this study
		*H. scrobiculatum*	simple-septa	simple-septa	Baird et al. 2013 [27]
		***H. squamulosum***	simple-septa	simple-septa	In this study
		*H. subsuccosum*	simple-septa	simple-septa	Baird et al. 2013 [27]
		***H. sulcatum***	simple-septa	simple-septa	In this study
		***H. yunnanense***	simple-septa	simple-septa	In this study
		***Hydnellum* sp 3**	simple-septa	simple-septa	In this study
		***Hydnellum* sp 4**	simple-septa	simple-septa	In this study
		***Hydnellum* sp 5**	simple-septa	simple-septa	In this study
	**Others**	*H. complicatum*	simple-septa	simple-septa	Baird et al. 2013 [27]
		*H. cumulatum*	simple-septa	simple-septa	Baird et al. 2013 [27]
		*H. lundellii*	simple-septa	simple-septa	Maas Geesteranus 1975 [1]
		*H. martioflavum*	simple-septa	simple-septa	Baird et al. 2013 [27]
**II**	***Subindufibulatum***	*H. caeruleum*	simple-septa, occasionally with clamp-connections	simple-septa	Baird & Khan 1986 [2]; In this study
		*H. ferrugipes*	simple-septa, occasionally with clamp-connections	simple-septa	Baird et al. 2013 [27]
		***H. fibulatum***	simple-septa, occasionally with clamp-connections	simple-septa	In this study
**III**	**Others**	*H. peckii*	mostly with clamp-connections, minority of simple-septa	mostly with clamp-connections, minority of simple-septa	Baird et al. 2013 [27]; In this study
		*H. versipelle*	mostly with clamp-connections, minority of simple-septa	mostly with clamp-connections, minority of simple-septa	Baird et al. 2013 [27]
**IV**	**Others**	*H. diabolus*	clamp-connections	simple-septa	Baird et al. 2013 [27]
**V**	***Hydnellum***	***H. atrospinosum***	clamp-connections	clamp-connections	In this study
		*H. suaveolens*	clamp-connections	clamp-connections	Baird et al. 2013 [27]
	***Caesispinosum***	*H. cyanopodium*	clamp-connections	clamp-connections	Baird 1986 [51]
		*H. scleropodium*	clamp-connections	clamp-connections	Harrison 1964 [33]
	**Others**	*H. geogenium*	clamp-connections	clamp-connections	Baird et al. 2013 [27]
	***Sarcodon***	*Sarcodon aspratus*	clamp-connections	clamp-connections	Maas Geesteranus 1971 [26]
		*S. imbricatus*	clamp-connections	clamp-connections	Baird et al. 2013 [27]
		*S. leucopus*	clamp-connections	clamp-connections	Mleczko et al. 2011 [66]
		*S. quercinofibulatus*	clamp-connections	clamp-connections	Pérez-De-Gregorio et al. 2011 [67]
		*S. scabripes*	clamp-connections	clamp-connections	Baird et al. 2013 [27]
		*S. squamosus*	clamp-connections	clamp-connections	Baird 1986 [34]
	**Others**	*H. regium*	hyphae with few simple-septa and with a few clamp-connections		Harrison 1964 [33]

## Data Availability

Publicly available datasets were analyzed in this study. All resulting alignments were deposited in TreeBASE (http://www.treebase.org (accessed on 17 August 2021); accession number S28676). All newly generated sequences were deposited in GenBank (https://www.ncbi.nlm.nih.gov/genbank/ (accessed on 23 September 2021); Table 1). All new taxa were deposited in Mycobank.

## Supplementary Materials

The following are available online at https://www.mdpi.com/article/10.3390/jof7100818/s1, Figure S1: Maximum likelihood tree illustrating the phylogeny of *Hydnellum* and *Sarcodon* based on ITS sequence dataset.
