# Supplementary material for: Multi-Gene Phylogeny and Taxonomy of Hydnellum (Bankeraceae, Basidiomycota) from China
Source: J Fungi (Basel). 2021 Sep 29;7(10):818. doi: 10.3390/jof7100818 (PMC8540476; doi:10.3390/jof7100818)
Supplement: Supplementary file 1 [file jof-07-00818-s001.zip › jof-1365812-supplementary.pdf]

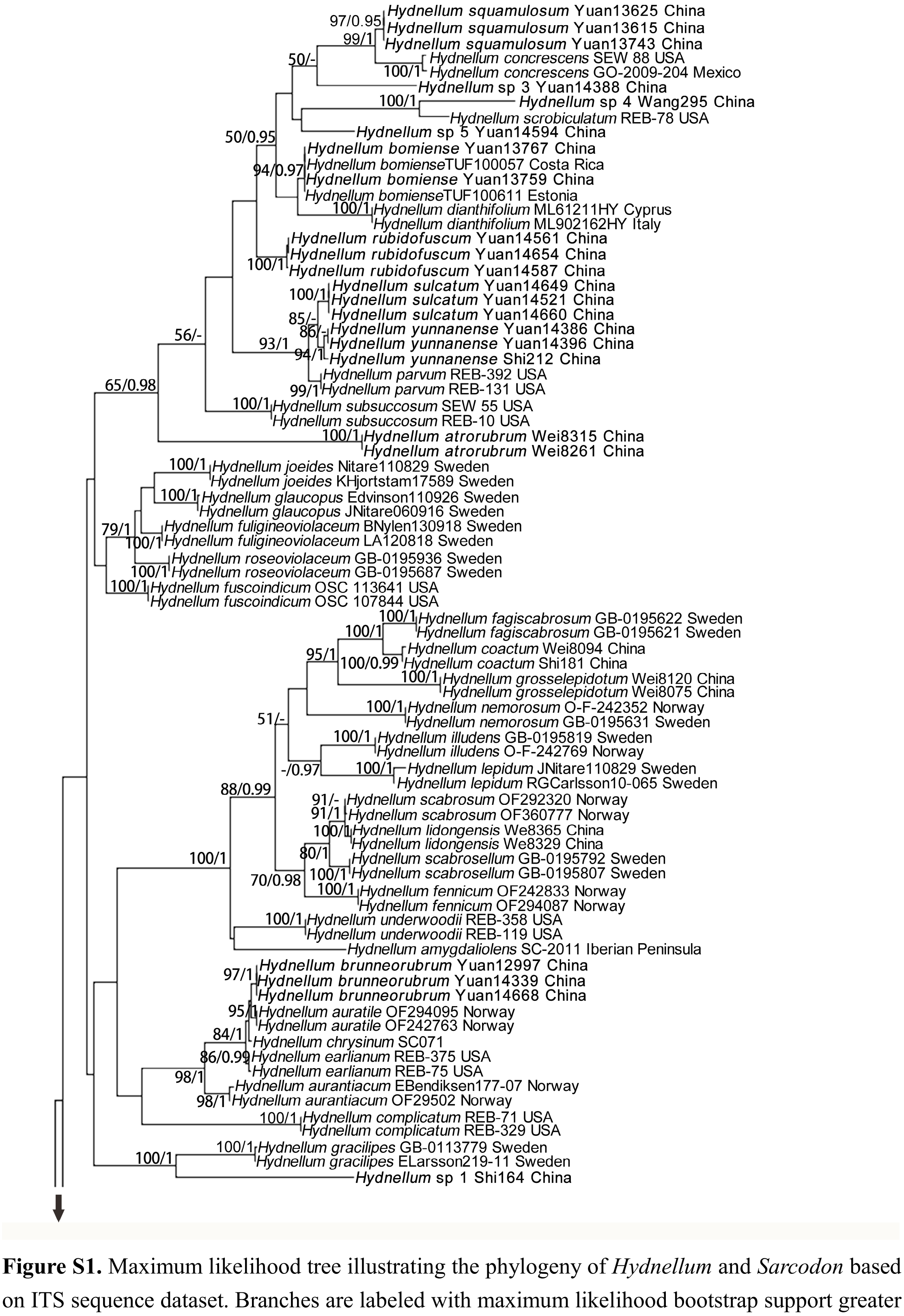

Figure S1. Maximum likelihood tree illustrating the phylogeny of *Hydnellum* and *Sarcodon* based on ITS sequence dataset. Branches are labeled with maximum likelihood bootstrap support greater than 50 % and Bayesian posterior probabilities greater than 0.95.

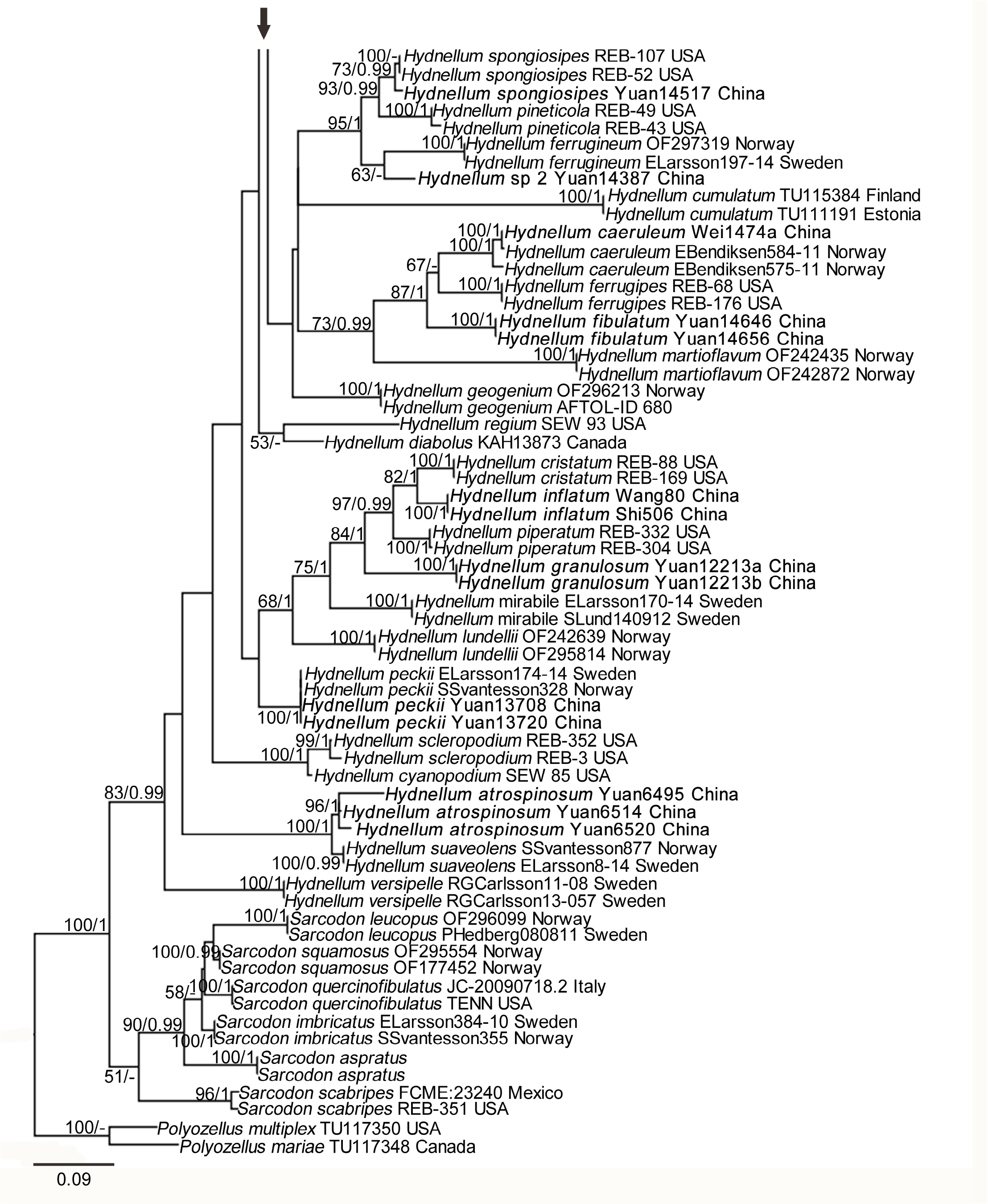

Figure S1. (continued)
